# Minimally invasive injection of biomimetic Nano@Microgel for in situ ovarian cancer treatment through enhanced photodynamic reactions and photothermal combined therapy

**DOI:** 10.1016/j.mtbio.2023.100663

**Published:** 2023-05-18

**Authors:** Xiaodong Ma, Wenhui Zhou, Rong Zhang, Cancan Zhang, Jiaqi Yan, Jing Feng, Jessica M. Rosenholm, Tingyan Shi, Xian Shen, Hongbo Zhang

**Affiliations:** a Joint Centre of Translational Medicine, The First Affiliated Hospital of Wenzhou Medical University, Wenzhou, 325000, China; b Department of General Surgery, The First Affiliated Hospital of Wenzhou Medical University, Wenzhou, 325000, China; c Ovarian Cancer Program, Department of Gynecologic Oncology, Zhongshan Hospital, Fudan University, Shanghai, 200032, China; d Southern Medical University Affiliated Fengxian Hospital, Shanghai, 201499, China; e Pharmaceutical Sciences Laboratory, Åbo Akademi University, Turku, 20520, Finland; f Turku Bioscience Centre, University of Turku and Åbo Akademi University, Turku, 20520, Finland; g Longgang District People's Hospital of Shenzhen, Shenzhen, 518172, China

**Keywords:** Gelatin methacryloyl, Photodynamic treatment, Photothermal treatment, Immunogenic cell death, Biomimetic Nano@Microgel

## Abstract

Photodynamic therapy (PDT) induces immunogenic cell death (ICD) by producing reactive oxygen species (ROS), making it an ideal method for cancer treatment. However, the extremely lower level of oxygen, short half-life of produced ROS, and limited photosensitizers accumulating in the tumor site via intravenous administration are the main reasons that limit the further application of PDT. To address these issues, we loaded the photosensitizer porphine (THPP) into biomimetic gold nanorod-mesoporous silica core-shell nanoparticles (Au-MSN NPs) to prepare Au@MSN/THPP@CM NPs. We then seeded the NPs together with catalase (CAT) into a gelatin methacryloyl (GelMA) microgel matrix to form Au@MSN-Ter/THPP@CM@GelMA/CAT microspheres consisting of biomimetic nano@microgel. The NPs and biomimetic nano@microgel exhibited enhanced photodynamic (PD) reaction and excellent photothermal conversion ability. Moreover, we further conjugated an endoplasmic reticulum (ER) targeting ligand Tosyl Ethylenediamine (Ter) on the surface of Au-MSN NPs. The results showed that both Au@MSN-Ter/THPP@CM NPs and the finally formed Au@MSN-Ter/THPP@CM@GelMA/CAT biomimetic nano@microgel induced precise and prolonged ER stress through photodynamic reactions, which stimulated the exposure of the proapoptotic calreticulin (CRT) on the cell membrane and increased the release of high mobility group box 1 (HMGB1) form the nucleus in SKOV3 cells under near-infrared (NIR) laser irradiation. Additionally, a single dose of the nano@microgel delivered through minimally invasive injection generated a significant anti-tumor effect in the SKOV3 cell line-derived orthotopic ovarian cancer mouse model through a PD and PT combination therapy. This study offers a new strategy for enhanced PDT and provides a PD/PT synergistic treatment method for ovarian cancer.

## Highlights

• An intratumorally injectable biomimetic nano@microgel for ovary cancer therapy is developed.
• The combination of enhanced photodynamic and photothermal therapy can be realized.
• The microgel exhibits sustained drug release and excellent tumor retention ability.
• The microgel can be used for ER targeted photosensitizer administration.
• The microgel can trigger ICD through enhanced photodynamic reactions.

## Graphical abstract

**Figure:**
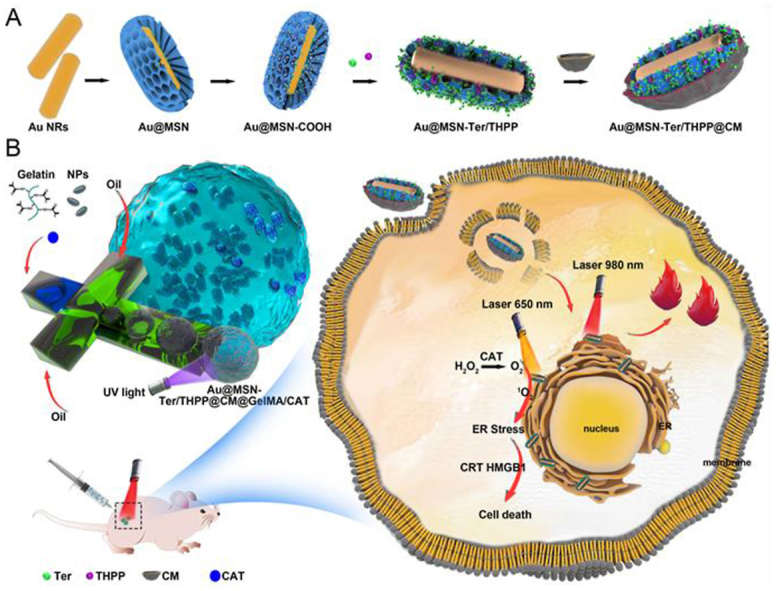

## 1 Introduction

Reactive oxygen species (ROS) are metabolic products produced by living cells and are often considered a double-edged sword [1,2]. Under normal conditions, ROS are mainly produced by mitochondria and endoplasmic reticulum (ER) and are utilized for various cellular activities, such as activating phagocytes [3]. However, high ROS accumulation in cells caused by pathological conditions or external stimuli can trigger irreversible damage and ultimately lead to cell death [4,5]. Prolonged cell stress, such as high levels of ROS accumulation in the ER (ER stress), can induce immunogenic cell death (ICD) or immunogenic apoptosis, which further activate the anti-tumor immune response [6,7]. This has inspired many researchers to develop ROS-inducing methods for removing unnecessary cells from the body, especially cancer cells. For instance, Zhang has developed ROS-inducing nanoplatforms that can be activated by ultrasound and have demonstrated excellent potential for breast cancer treatment in vitro and in vivo [8]. Similarly, Gang has developed ROS-inducing nanozymes, showing great potential for breast cancer therapy [9]. Therefore, ROS-inducing has emerged as a promising strategy for cancer therapy.

Photodynamic therapy (PDT) is a promising topical treatment for ROS-based cancer therapy that uses singlet oxygen generated by excited photosensitizers under an appropriate light source to induce cell death [[10], [11], [12]]. However, the oxygen deficiency characteristic of tumors limits the ROS production by traditional photosensitizers administration. To address this issue, strategies have been developed to generate oxygen in situ or deliver exogenous oxygen. Generating oxygen in situ is a more reliable method for enhancing the PDT effect compared to unstable exogenous oxygen delivery. Recently, a reagent that catalyzes hydrogen peroxide (H_2_O_2_) to produce oxygen (O_2_) has been used to enhance PDT because the concentration of H_2_O_2_ is much higher than that of superoxide anion (O_2_-) in cancer cells [13]. For instance, a dual nanozyme Au_2_Pt-PEG-Ce6 has been shown to have catalase activity, which generates O_2_ in cancer cells and has enhanced anti-tumor activity in cervical cancer models when combined with PDT therapy [14]. Encapsulating catalase (CAT) in temperature-dependent nanoformation RGD-BPNS@SMFN has achieved enhanced PDT effect and promising synergistic antitumor effects with photothermal therapy (PTT) [15]. Furthermore, CAT-loaded and HER-2 nanobody-conjugated nanoparticles have shown tremendous potential for ovarian cancer therapy [16]. Therefore, designing materials with catalase or catalase-like activity can be a useful strategy for enhancing the PDT effect.

However, the traditional method for administering photosensitizers is intravenous injection, which can result in uneven distribution throughout the body and increase metabolic stress. Furthermore, only a small amount of photosensitizers penetrating the tumor tissue can lead to limited therapeutic effects [17]. Over the past few decades, nano-delivery systems have become ideal vehicles for delivering functional cargoes, such as DNA, RNA, proteins, and small molecules, targeted to the tumor area due to their flexible structure design, excellent cargo loading capacity, and enhanced permeability and retention effect (EPR). Among these systems, biomimetic-designed nanoparticles, such as those designed to mimic cell surfaces, have attracted widespread interest for targeted intracellular drug delivery due to their excellent biocompatibility and targeting ability [[18], [19], [20]]. For instance, engineered cancer cell membrane-coated CLip-PC@CO-LC NPs have been shown to have great potential for combined RNAi and chemotherapy in the treatment of lung cancer [21]. Similarly, mesenchymal stem cell-derived nanoparticles containing Notch-1 suppressor have demonstrated significant potential for promoting hypoxia and inhibiting angiogenesis in vitro and in vivo [22]. Furthermore, the extensive homology and homing ability of cell camouflaging nanoparticles make them more easily hidden from the immune system and able to reach their target destination [[23], [24], [25]]. Therefore, cell camouflaging nanoparticles may offer a promising solution to address photosensitizer target loss during intravenous administration.

Inspired by our previous work, we have demonstrated that mesoporous silica-coated gold nanorod nanoparticles (Au@MSN NPs) have a wide range of applications in multi-drug delivery and PTT-based dual anti-tumor therapy [26]. In this study, we utilized Au@MSN NPs as a vehicle for intracellular delivery of a photosensitizer, Tetrakis (4-hydroxyphenyl)-porphine (THPP), to achieve PTT and PDT combined therapy using only one nanocarrier. However, the efficacy of PDT is limited by the short lifetime of ROS, particularly singlet oxygen, generated by the photosensitizer during PDT in biological systems. Additionally, the diffusion distance of ROS is limited to only 0.01–0.02 ​μm in biological systems, which means the location of ROS production affects its therapeutic effect [27]. Therefore, the precise transportation of photosensitizers to important or fragile organelles, such as the endoplasmic reticulum (ER), allows the ROS produced by photosensitizers to accurately attack the "lethal sites" of cancer cells and achieve better therapeutic effects [28]. We conjugated an ER targeting ligand, Tosyl Ethylenediamine (Ter), on the surface of Au@MSN NPs to develop a more precise photosensitizer nano-delivery system for cancer treatment. Once the NPs enter the cancer cells, the Ter ligand can enable the THPP-loaded NPs to precisely move to the ER. Under laser irradiation, the constantly produced ROS will induce irreversible ER stress and activate the ICD. However, photosensitizers' accumulation in the tumor area by nanocarriers is limited due to nanoparticles' excretion in the kidney and preferred accumulation in the liver, requiring multiple intravenous administration of large doses to achieve long-term PDT therapeutic effects. Local administration of photosensitizers is a better method because the photosensitizers can directly reach the tumor site without relying on blood circulation, resulting in reduced side effects and higher efficiency [29]. Gelatin methacryloyl (GelMA) microgel, a gelatin derivative, is an ideal candidate for local administration of photosensitizers due to its good biocompatibility, good retention ability, and ability to cross-link by UV [30]. Therefore, we loaded our developed ER-targeting and PDT-generating biomimetic gold nanorod-mesoporous silica core-shell nanoparticles (Au@MSN-Ter/THPP@CM NPs) with CAT into the GelMA microgel on a microfluidic chip to form Au@MSN-Ter/THPP@CM@GelMA/CAT microspheres consisting of biomimetic nano@microgel. We expect this will enable more efficient administration of photosensitizers and enhanced PD reactions for cancer therapy.

Ovarian cancer remains one of the most malignant cancers affecting women, and effective treatments are needed urgently [[31], [32], [33]]. In order to develop a more effective photodynamic therapy (PDT) method for ovarian cancer, we evaluated the anti-tumor activity of our developed biomimetic nano@microgel systems, including Au@MSN-Ter/THPP@CM NPs and Au@MSN-Ter/THPP@CM@GelMA/CAT nano@microgel, in both in vitro and in vivo ovarian cancer models (Schematic Figure). The Au@MSN-Ter/THPP@CM NPs showed excellent photothermal conversion ability and were able to be taken up efficiently by ovarian cancer cells. We tested the anti-tumor activity of both Au@MSN-Ter/THPP@CM NPs and Au@MSN-Ter/THPP@CM@GelMA/CAT biomimetic nano@microgel, and found that both exhibited significant cell proliferation inhibition and cell death induction in ovarian cancer cells. Furthermore, we verified the design of CAT for oxygen production and ER-targeted THPP delivery, which led to more precise and enhanced PDT reactions in SKOV3 cells. Finally, in an orthotopic ovarian cancer mouse model, our newly designed Au@MSN-Ter/THPP@CM@GelMA/CAT microgel were able to provoke two ICD markers and demonstrated significant anti-tumor activity through a combination of PDT and photothermal therapy (PTT). Overall, our study suggests that the Au@MSN-Ter/THPP@CM@GelMA/CAT biomimetic nano@microgel may be a promising candidate for enhanced PDT and PTT combined therapy for ovarian cancer.

**Schematic Figure:**
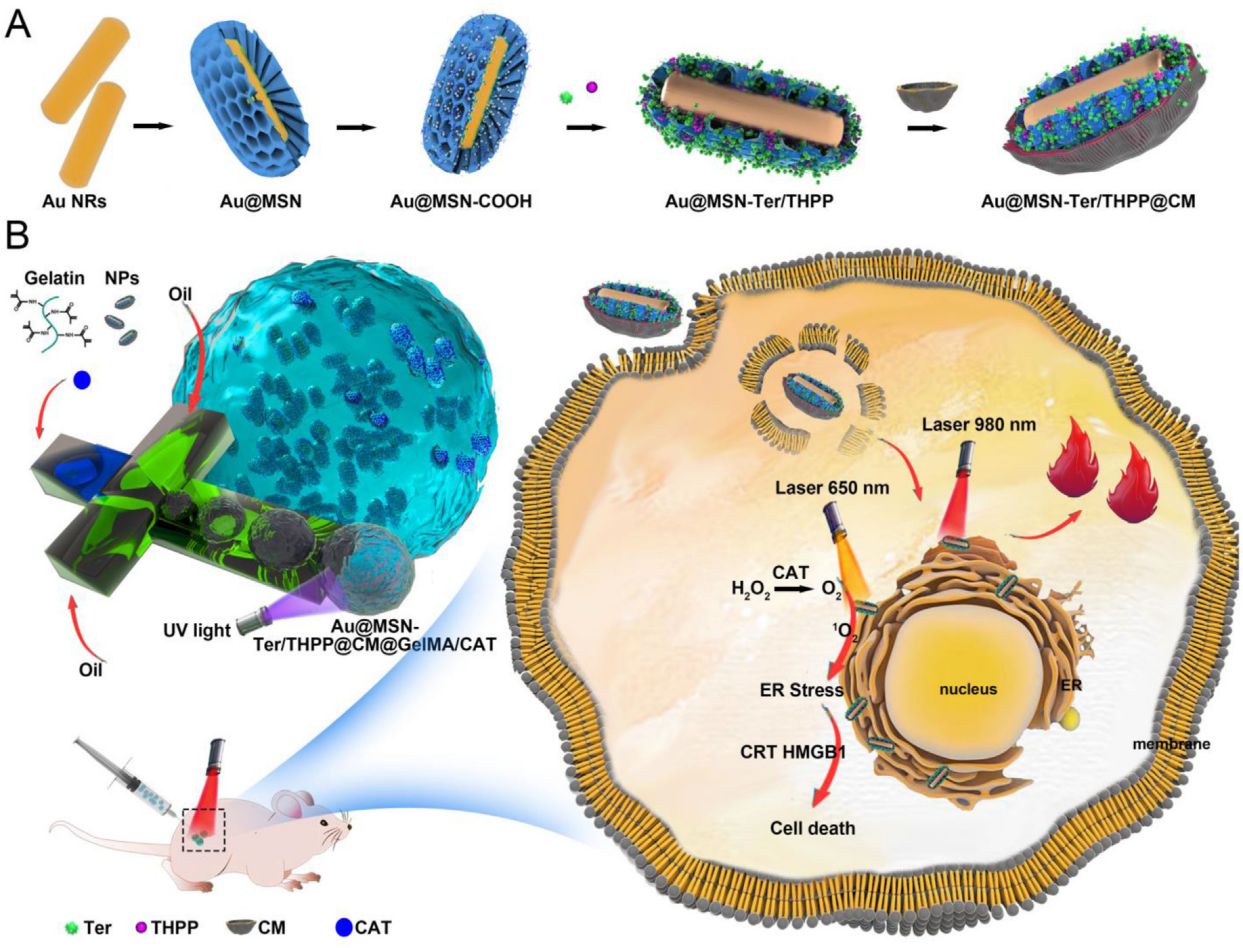
The schematic of Au@MSN-Ter/THPP@CM NPs and Au@MSN-Ter/THPP@CM@GelMA/CAT microsphere preparation and working model.

## 2 Materials and methods

### 2.1 Materials

Tetrakis (4-hydroxyphenyl)-porphine (THPP), Tosyl Ethylenediamine (TER), N-(3-dimethylaminopropyl)-N-ethylcarbodiimide hydrochloride (EDC·HCl), N-hydroxysuccinimide (NHS), 2-Hydroxy-4'-(2-hydroxyethoxy)-2-methylpropiophenone and Methacrylic anhydride were purchased from Tansoole (Shanghai, China). Sodium borohydride (NaBH_4_), tetraethyl orthosilicate (TEOS), silver nitrate (AgNO_3_), l-ascorbic acid, tetrachloroauric acid (HAuCl_4_), cetyltrimethyl ammonium bromide (CTAB), succinic anhydride and Mineral oil were purchased from Sigma-Aldrich (Burlington, MA, US). Gelatin was purchased from Aamas (Shanghai, China).

### 2.2 AuNRs synthesis

The synthesis procedure for AuNRs followed the methodology outlined in our previous work. Initially, a balloon flask was used to mix 7.5 ​mL of 0.1 ​M CTAB solution and 0.25 ​mL of 0.01 ​M HAuCl_4_ solution. The mixture was stirred for 5 ​min before the addition of 0.6 ​mL of 0.01 ​M cold NaBH_4_ solution with vigorous stirring. Four hours later, a separate beaker was used to mix 20 ​mL of 0.01 ​M HAuCl_4_, 4.8 ​mL of 0.01 ​M AgNO_3_, 400 ​mL of 0.1 ​M CTAB, and 7.6 ​mL of 1 ​M HCl, which was stirred for 10 ​min at 30 ​°C. The resulting mixture was then quickly added to the stirring balloon flask along with 4 ​mL of 0.1 ​M ascorbic acid. Subsequently, 0.96 ​mL of the prepared gold seeds solution was added to the flask and left overnight. Finally, the AuNRs were collected by centrifugation and washed with deionized water.

### 2.3 Au@MSN-NH_2_ and Au@MSN-COOH NPs synthesis

The collected AuNRs were firstly dispersed in deionized water and added to 50 ​mL of 1 ​mM CTAB aqueous solution. Next, the pH of the mixture was adjusted to pH 10–11 with 0.1 ​M NaOH, and then 1.4 ​mL of 20% (v/v) TEOS ethanol solution was added. After continuous stirring at 30 ​°C overnight, Au@MSN NPs were collected by centrifuge and washed with 0.6% (w/v) NH_4_NO_3_ ethanol solution. Finally, Au@MSN NPs were dispersed overnight in APTES containing ethanol solution to get the surface-activated Au@MSN-NH_2_ NPs. And Au@MSN-NH_2_ NPs were further dispersed in succinic anhydride containing DMF solution overnight under stirring to obtain Au@MSN-COOH NPs.

### 2.4 Synthesis of Au@MSN-Ter NPs and THPP loading

To prepare Au@MSN-Ter NPs, a mixture of Au@MSN-COOH NPs, Ter, NHS, and EDCI was stirred in DMF at room temperature overnight. The resulting mixture was then collected by centrifugation, and washed with DMF to obtain the Au@MSN-Ter NPs. Subsequently, the Au@MSN-Ter NPs were stirred with a THPP DMF solution overnight to complete the THPP loading process.

### 2.5 Cell culture

SKOV3 and NHDF cell lines were purchased from ATCC, and SKOV3 was cultured in 10% FBS and 1% PS containing McCoy's 5a Medium. NHDF cells were cultured in 10% FBS and 1% PS containing DMEM Medium. The OVCAR3 cell line was gifted from the laboratory of Research Program in Systems Oncology at the University of Helsinki and cultured in 10% FBS, 1% PS, and 0.01 ​mg/mL bovine insulin containing RPMI-1640 Medium. All cells were maintained in the incubator of 37 ​°C and 5% CO_2_ atmosphere.

### 2.6 Cancer cell membrane extraction and NPs coating

SKOV3 cells were collected and washed three times with PBS and then incubated in tris buffer (containing 10 ​mM tris, 10 ​mM MgCl_2_ and protease inhibitor) for 1 ​h at 4 ​°C. After that, the cells were sonicated for 10 ​min in an ice bath, and cell membrane fragments were obtained by differential centrifugation (500 ​g for 10 ​min, 10,000 ​g for 10 ​min). Then the aqueous solution was frozen dried and stored at −20 ​°C for further use. Next, the cancer cell membrane was mixed with different NPs in deionized water at 4 ​°C and stirred overnight. Finally, the cell membrane coated Au@MSN-Ter/THPP@CM and Au@MSN/THPP@CM NPs were obtained by centrifuge.

### 2.7 GelMA, Au@MSN-Ter/THPP@CM@GelMA and Au@MSN-Ter/THPP@CM@GelMA/CAT microgel preparation

Firstly, 20 ​g of gelatin was dissolved in 200 ​mL PBS at 60 ​°C. Next, 16 ​mL of MA was slowly pumped into the gelatin solution with a micro syringe pump at 0.25 ​mL/min. Two hours later, 200 ​mL PBS was added to terminate the reaction for 15 ​min. After that, the prepared GelMA solution was dialyzed (3500 ​MWCO dialysis bag) overnight at 38 ​°C. And then, the GelMA solution was centrifuged at 37 ​°C to remove the insoluble and continued dialysis for another 2 days. Finally, the prepared GelMA was frozen dried and stored in −80 ​°C.

The Au@MSN-Ter/THPP@CM@GelMA and Au@MSN-Ter/THPP@CM@GelMA/CAT microgel were prepared in a microfluidic chip, which contains two different channels: internal flow was the mixture of 150 ​mg/mL GelMA, 10 ​mg/mL photocrosslinker, 5 ​mg/mL CAT and 10 ​mg/mL Au@MSN-Ter/THPP@CM; while the external flow contains of 5% Span 80 in mineral oil. And the Au@MSN-Ter/THPP@CM@GelMA and Au@MSN-Ter/THPP@CM@GelMA/CAT microspheres were produced with the external and internal flow rate of 12–1:1 and cured by UV light. Finally, the Au@MSN-Ter/THPP@CM@GelMA and Au@MSN-Ter/THPP@CM@GelMA/CAT microgel were collected by centrifuge and washed with 75% ethanol.

### 2.8 Cytotoxicity assay

SKOV3 and OVCAR3 cells were seeded at the density of 5000 per well of 96 well plate. The next day, different NPs formulations were added at indicated concentrations. 12 ​h later, the cells were selectively irradiated with 1 ​W/cm^2^ of 980 ​nm laser for 10 ​min and followed 0.4 ​W/cm^2^ of 650 ​nm laser for 5 ​min. And after 24 ​h, 10 μl of WST-1 regent per well were added to test the cell viability.

### 2.9 IF staining assay

The cells were first co-incubated with different NPs formulations for indicated times and selectively treated with 0.4 ​W/cm^2^ of 650 ​nm laser for 5 ​min. 6 ​h later, the cells were washed with PBS and fixed with 4% PFA for 5 ​min. And then, the cells were washed with PBS three times and incubated with 1:500 diluted *anti*-CRT (Abcam) or *anti*-HMGB1 (Abcam) dilutions for 1 ​h at room temperature in dark. Next, cells were washed with PBS three times and stained with 5 ​mg/mL DAPI for 5 ​min. Finally, the images were captured by confocal microscope and analyzed by image J.

### 2.10 ROS detection

The cells were first co-incubated with Au@MSN-Ter/THPP@CM@GelMA/CAT or Au@MSN-Ter/THPP@CM@GelMA microgel for 48 ​h. After that, the cells were incubated with the reactive oxygen test dye DCFH-DA (10 ​μM) for 20 ​min, and followed irradiate with 0.4 ​W/cm^2^ of 650 ​nm laser for 5 ​min. Finally, the cell nuclei were labeled with Hochest33342 and were observed by confocal microscope.

### 2.11 Cellular uptake, lysosomal escape and ER targeting assay

Au@MSN-Ter/THPP@CM and Au@MSN/THPP@CM NPs were used for cellular uptake. While fluoresce dye Cy5.5 was first labeled on the surface of Au@MSN-Ter@CM NPs, and then the cancer cells were incubated with Cy5.5 labeled NPs for indicated times. Next, ER-Tracker (1 ​μM) or lysosomal tracker was added to the culture medium and incubated for 30–120 ​min at 37 ​°C according to the protocol. After that, the cells were fixed with 4% PFA, washed and stained with DAPI for 5 ​min. Finally, the cells were observed by confocal microscope.

### 2.12 Calcein-AM and PI staining assay

Different formulations of Au@MSN-Ter/THPP@CM@GelMA/CAT and Au@MSN-Ter/THPP@CM@GelMA were firstly incubated with SKOV3 and OVCAR3 cells for 48 ​h, and then selectively treated with lasers according to the corresponding groups. 6 ​h later, Calcein-AM (2 ​μM) and PI (4.5 ​μM) were added to the culture medium and incubated for 20 ​min. The cells were observed by confocal microscope.

### 2.13 Flow cytometry assay

SKOV3 and OVCAR3 cells were firstly incubated with Au@MSN-Ter/THPP@CM@GelMA/CAT or Au@MSN-Ter/THPP@CM@GelMA for 48 ​h, and then selectively treated with lasers. 6 ​h later, cells were harvested and stained with Annexin V and PI according to the instructions of the kit, and the cells were analyzed by a flow cytometer.

### 2.14 Animal study

All animal experiments and operations followed the guideline and approved by the Animal Research Committee of Southern Medical University affiliated Fengxian Hospital, China (SHZY-2021102601). The animal study was performed with ovarian cancer orthotopic xenograft mouse models. Briefly, 2 ​× ​10^5^ SKOV3 cells in PBS were first injected into the axillary fat pad of 6 weeks old female nude mice. The primary SKOV3 tumors were surgically removed and cut into 3–4 ​mm small cubes under a cryogenic environment once the tumors reached to 2 ​cm diameter. Next, the small tumor cubes were further surgical placed into the ovarian tissues of 6 weeks old female nude mice.

One week late, the mice were randomly assigned into four groups (each group ≥6). Group A: injected with 100 ​μL of PBS from tail vain; Group B: injected with 100 ​μL of Au@MSN-Ter/THPP@CM NPs from tail vain; Group C and D: 30 ​μL of Au@MSN-Ter/THPP@CM@GelMA/CAT microgel were surgical injected into the tumor site once. The amount of Au@MSN-Ter/THPP@CM NPs and Au@MSN-Ter/THPP@CM@GelMA/CAT used for the animal study equals 2.5 ​mg/kg THPP. On the next day of different formulations injections, mice in Group A, B and C were first treated with 1 ​W/cm^2^ of 980 ​nm laser irradiation for 10 ​min, and followed by 0.4 ​W/cm^2^ of 650 ​nm laser irradiation for another 5 ​min at the tumor area. The mice continued treated with lasers once each three days, and the mice were sacrificed 15 days late. The main organs (including heart, liver, spleen, lung, and kidney) and tumor tissues were collected for further HE and IHC staining.

### 2.15 Statistical analysis

Both the quantified and none quantified data were collected from triplicated independent experiments. The confocal images were quantified by using the Image J software. The data analysis and graphical work were performed with GraphPad and SPSS 20.0 software. P＜0.05 was considered significant.

## 3 Results

### 3.1 Au@MSN and Au@MSN-Ter/THPP@CM NPs preparation and characterization

**Fig. 1:**
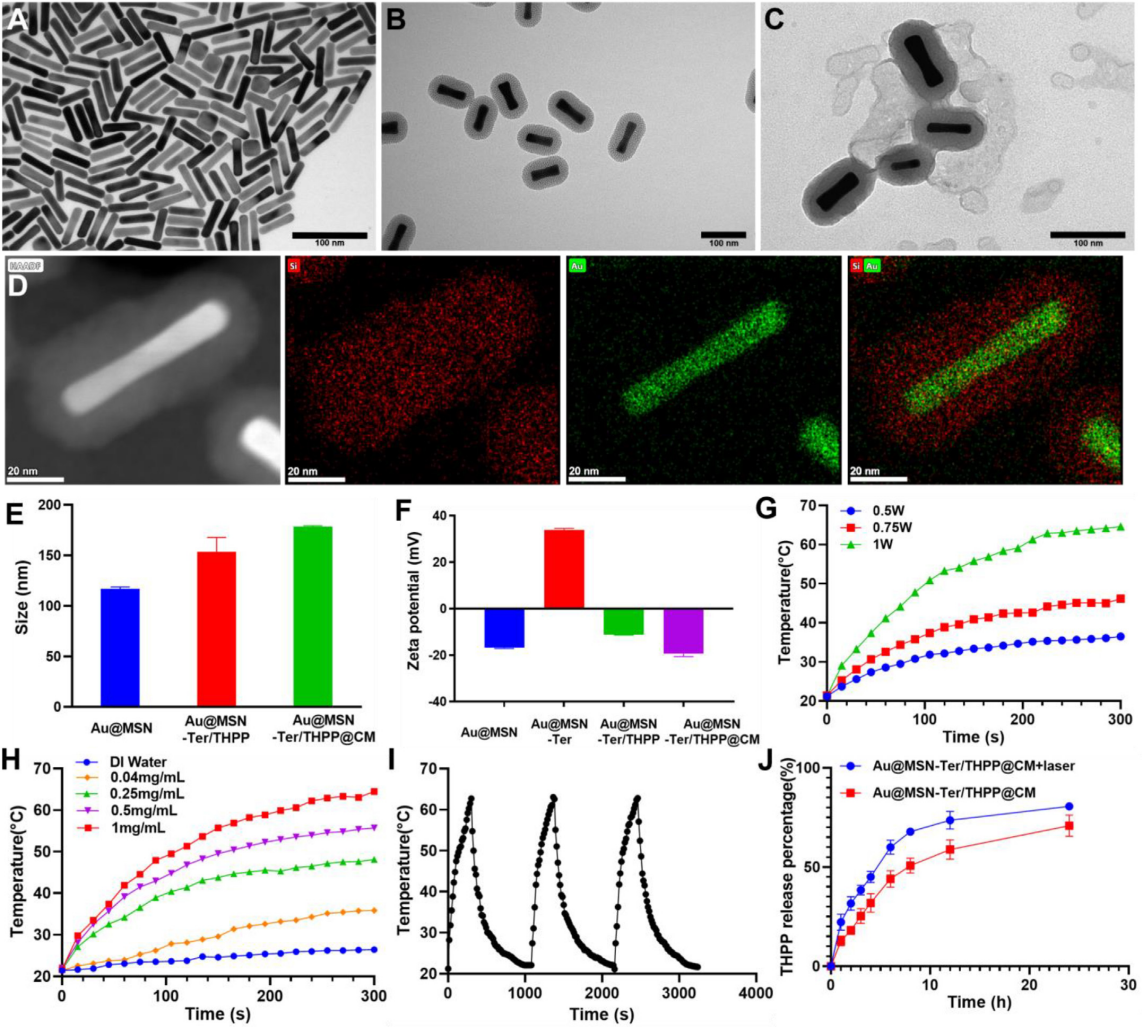
Morphologies, photothermal effect and drug releasing of different NPs. A-C. TEM images of Au NRs, Au@MSN NPs, Au@MSN-Ter/THPP@CM NPs (Scale bar ​= ​100 ​nm); D. Image of high-angle annular dark-field and elemental mapping of Au@MSN NPs (Scale bar ​= ​20 ​nm); Particle size (E), Zeta-potential (F) and photothermal response of Au@MSN-Ter/THPP@CM NPs under 980 ​nm laser irradiation (G–I) (Total concentration of nanoparticles is used); J. THPP releasing from Au@MSN-Ter/THPP@CM NPs in PBS buffer with or without laser (980 ​nm, 1 ​W/cm^2^, 10 ​min).

As we have described before, the Au@MSN NPs were prepared by two steps of seed mediated growth methods [26]. Subsequently, a small molecule Tosyl Ethylenediamine (Ter), which can be used for ER targeting was conjugated on the surface of aminated Au@MSN NPs via a linker, succinic anhydride. After washing, the THPP was further loaded into the Au@MSN-Ter NPs by overnight incubation in DMF to form the Au@MSN-Ter/THPP NPs. Finally, the cell membrane extracted from Ovarian cancer SKOV3 cells was coated on to the NPs to form the biomimetic NPs Au@MSN-Ter/THPP@CM. As the TEM results shown in Fig. 1 A, the naked gold nanorods were evenly distributed in an aqueous solution with the size of 56.1 ​± ​5.3 ​nm. While after mesoporous silica layer growing on the surface of gold nanorods (Fig. 1 B) and both THPP and Ter conjugating, the size of Au@MSN-Ter/THPP NPs and Au@MSN-Ter/THPP@CM NPs (Fig. 1C) were dramatically increased to 153.5 ​± ​14.3 ​nm and 178.6 ​± ​0.8 ​nm respectively (Fig. 1 E). The elemental mapping result of Au@MSN NPs in Fig. 1 D and Supplementary Fig. 1 also showed that Si tightly surrounded the Au. And after conjugating of Ter ligand on the surface of Au@MSN NPs, the Fourier transform IR analyzing result (FTIR, Supplementary Fig. 2) showed that the amino group on Ter disappears in Au@MSN-Ter NPs, there came a new peak at 1631 ​cm^−1^, which was caused by new introduced amido link. In addition, Zeta potential of Au@MSN NPs changed from −16.7 ​± ​0.35 to 33.8 ​± ​0.6 after Ter modification, and Zeta potential changed to −11.1 ​± ​0.32 after loading with THPP due to the excess hydroxyl group on THPP. These results demonstrate the success of Au@MSN-Ter synthesis and THPP loading Moreover, after THPP loading and cell membrane coating, the original outlayer pore structure of Au@MSN NPs was coated with a condensed outlayer, and zeta potential of the corresponding nanoparticles also changed to −19.4 ​± ​1.2 which illustrate the successful wrapping of cancer cell membranes. (Fig. 1C, F). Under the protection of cancer cell membrane, Au@MSN-Ter/THPP@CM NPs have good stability. When the nanoparticles were dispersed in the medium, the particle size remained stable within 10 days (Supplementary Fig. 3).

Subsequently, we tested the photothermal activity and singlet oxygen production of the prepared Au@MSN-Ter/THPP@CM NPs. SOSG was used as a probe to detect the generation of singlet oxygen. From Supplementary Fig. 4, under the 650 ​nm laser irradiation, SOSG fluorescence intensities of THPP group and Au@MSN-Ter/THPP@CM NPs group increased over time, the increase in NPs group is lower than that of THPP group, which might be due to the stacking of THPP in MSN. Photothermal activity of Au@MSN-Ter/THPP@CM NPs shown that, the temperature of 1 ​mg/mL NPs solution dramatically increased up to 64 ​°C by 1 ​W/cm^2^ 980 ​nm laser irradiation for only 5 ​min, while only increased up to 36 ​°C under 0.5 ​W/cm^2^ 980 ​nm laser irradiation (Fig. 1 G). And once the concentration of NPs decreased from 1 ​mg/mL to 0.5 ​mg/mL and 0.25 ​mg/mL, the temperature increased to 55 ​°C and 48 ​°C respectively by 1 ​W/cm^2^ 980 ​nm laser irradiation for 5 ​min (Fig. 1H). In addition, the NPs also had excellent photothermal stability, because there was no obvious temperature change happened in three repeated photothermal cycles. These results indicated that the excellent photothermal conversion ability of Au@MSN-Ter/THPP@CM NPs could be activated by 980 ​nm laser irradiation, and affected by NPs’ concentration and irradiation time. Finally, we further evaluated THPP delivery efficiency of Au@MSN-Ter/THPP@CM NPs by the loading and releasing THPP from Au@MSN-Ter/THPP@CM NPs. As the results detected by a UV-Spectrophotometer, the THPP loading capacity and loading efficiency of Au@MSN-Ter/THPP@CM NPs were 11.5% and 73.2%. And as shown in Fig. 1 J, THPP releasing from Au@MSN-Ter/THPP@CM NPs was time-dependent and could reach 50.8 ​± ​3.8% at 8 ​h in 0.5% Tween 80 ​PBS buffer. While the total releasing of THPP was significantly (p＜0.001) increased up to 67.9 ​± ​1.8% under 980 ​nm laser (1 ​W/cm^2^) irradiation at 8 ​h time point (980 ​nm laser was treated before each time point). These results indicated that the Au@MSN-Ter/THPP@CM NPs had good THPP loading capability and sustained THPP releasing manner.

### 3.2 Cytotoxicity and cellular uptake of Au@MSN-Ter/THPP@CM NPs in ovarian cancer cells

**Fig. 2:**
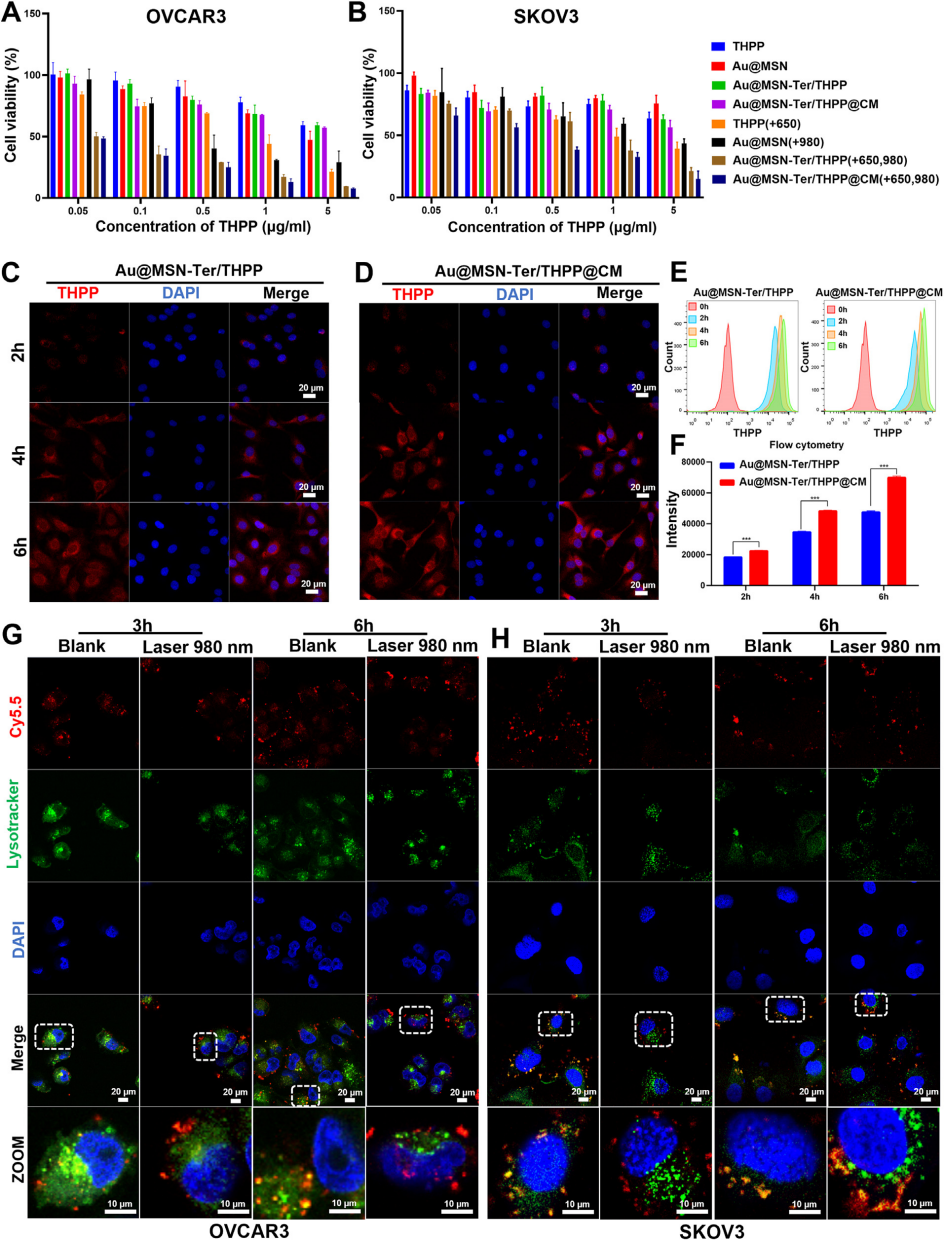
Cytotoxicity and cellular uptaking of different NPs. OVCAR3 (A) and SKOV3 (B) cells viability after different concentrations of various NPs for 24 ​h following with 650 ​nm laser (0.4 ​W/cm^2^, 5 ​min) and/or 980 ​nm laser (1 ​W/cm^2^, 10 ​min) irradiation. Confocal microscope images (C and D) and flow cytometry analysis (E and F) of various NPs' cellular uptaking after 2–6 ​h co-incubation (Scale bar: 20 ​μm). G and H. Confocal microscope images of Cy5.5 labeled Au@MSN-Ter@CM NPs' lysosome escape after 3–6 ​h co-incubation and selectively treated with980 nm laser (1 ​W/cm^2^, 10 ​min) irradiation. (Red: Cy5.5; Green: lysotracker; Blue: DAPI). (For interpretation of the references to color in this figure legend, the reader is referred to the Web version of this article.)

We have verified the excellent photothermal conversion ability and sustained THPP release of Au@MSN-Ter/THPP@CM NPs, to further evaluate the inhibitory effect of different preparations on different ovarian cancer cells. We performed WST-1, flow cytometry and confocal microscopy assays to evaluate the cytotoxicity and cellular uptaking of Au@MSN-Ter/THPP@CM NPs in Ovarian cancer cell lines OVCAR3 and SKOV3. As shown in Fig. 2 A and B, the cells viability was analyzed by WST1 assays after the cells were co-incubated with different NP formulations for 24 ​h and selectively treated with 980 ​nm (1 ​W/cm^2^, 10 ​min) and 650 ​nm (0.4 ​W/cm^2^, 5 ​min) lasers irradiation. It was shown that, in Au@MSN NPs groups, the cell viabilities in both OVCAR3 and SKOV3 cells were significantly decreased after 980 ​nm laser treatment, especially in the highest NPs concentration groups, which is attributed to the photothermal effect induced cell death. And for 5 ​μg/mL Au@MSN-Ter/THPP@CM NPs, 650 ​nm laser induced obvious cell viability change in OVCAR3 and SKOV3 cells. These results indicated that the Au@MSN-Ter/THPP@CM NPs can generate lethal level photodynamic reactions under 650 ​nm laser irradiation, and the Au@MSN-Ter/THPP@CM NPs have great potential for improved PDT. In addition, the cell viabilities of Au@MSN-Ter/THPP@CM NPs treated with both 980 ​nm and 650 ​nm lasers, had more significant cell viability change in both OVCAR3 and SKOV3 cells compared to single laser treatment, which were 7.7 ​± ​0.5% and 14.8 ​± ​6.4%, respectively. While, in normal human dermal fibroblasts NHDF cells (Supplementary Fig. 5), the cell viabilities of 5 ​μg/mL Au@MSN-Ter/THPP@CM NPs treated with both 980 ​nm and 650 ​nm lasers was 41.5 ​± ​2.8%, this indicated that the Au@MSN-Ter/THPP@CM NPs had tumor cell selectivity at some extent.

Next, we further utilized flow cytometry assay and confocal microscope to evaluate the intracellular behavior of Au@MSN-Ter/THPP and Au@MSN-Ter/THPP@CM NPs. As shown in Fig. 2, both confocal microscope imaging (Fig. 2C and D) and flow cytometry analyzing results (Fig. 2 E and F) showed that the red THPP fluorescent signal represented Au@MSN-Ter/THPP and Au@MSN-Ter/THPP@CM NPs gathered in SKOV3 cells after only 2 ​h co-incubation. We also noticed that, the red fluorescent positive cells and fluorescent intensity per cell were gradually increased by time in Au@MSN-Ter/THPP@CM NPs treated groups. And compared to SKOV3 cells (Supplementary Fig. 7), the red THPP fluorescent signal in OVCAR3 and NHDF cells (Supplementary Fig. 8) were less. No difference in THPP positive cells between Au@MSN-Ter/THPP and Au@MSN-Ter/THPP@CM NPs treated NHDF (Supplementary Fig. 9). Since the NPs were coated with SKOV3 cell membrane, these results indicated that the SKOV3 cell membrane coating can improve the selective cell uptake into SKOV3 cells but not other cells. Subsequently, we tested the endo/lysosome escaping ability, which is one of the crucial determinants for successful intracellular delivery. Firstly, Cy5.5 labeled Au@MSN-Ter@CM NPs were co-incubated with OVCAR3 and SKOV3 cell for scheduled 3 and 6 ​h, and then the cells were selectively treated with laser 980 ​nm for 10 ​min in the prior of green lysotracker and blue DAPI staining. As shown in Fig. 2 G and H, the free red fluorescent signal, which represented free Au@MSN-Ter/Cy5.5@CM NPs, were abundantly detected in both cells after 3 ​h and 6 ​h co-incubation, while most of the red fluorescent signal were merged with green fluorescence signal, which represented lysosome, except for OVCAR3 cells at 3 ​h’ time point. These results mean that the Au@MSN-Ter/THPP@CM NPs were taken by the cells, but mass of Au@MSN-Ter/THPP@CM NPs were inside of the lysosomes. While in the laser treated groups, the red and green fluorescence signals were obviously separated in both OVCAR3 and SKOV3 cells at 3 and 6 ​h. This result is similarly with our previous finding and confirmed the endo/lysosome escape of Au@MSN-Ter/THPP@CM NPs, as stimulated by PTT [26].

### 3.3 Evaluate the ICD provoking ability of Au@MSN-Ter/THPP@CM NPs

**Fig. 3:**
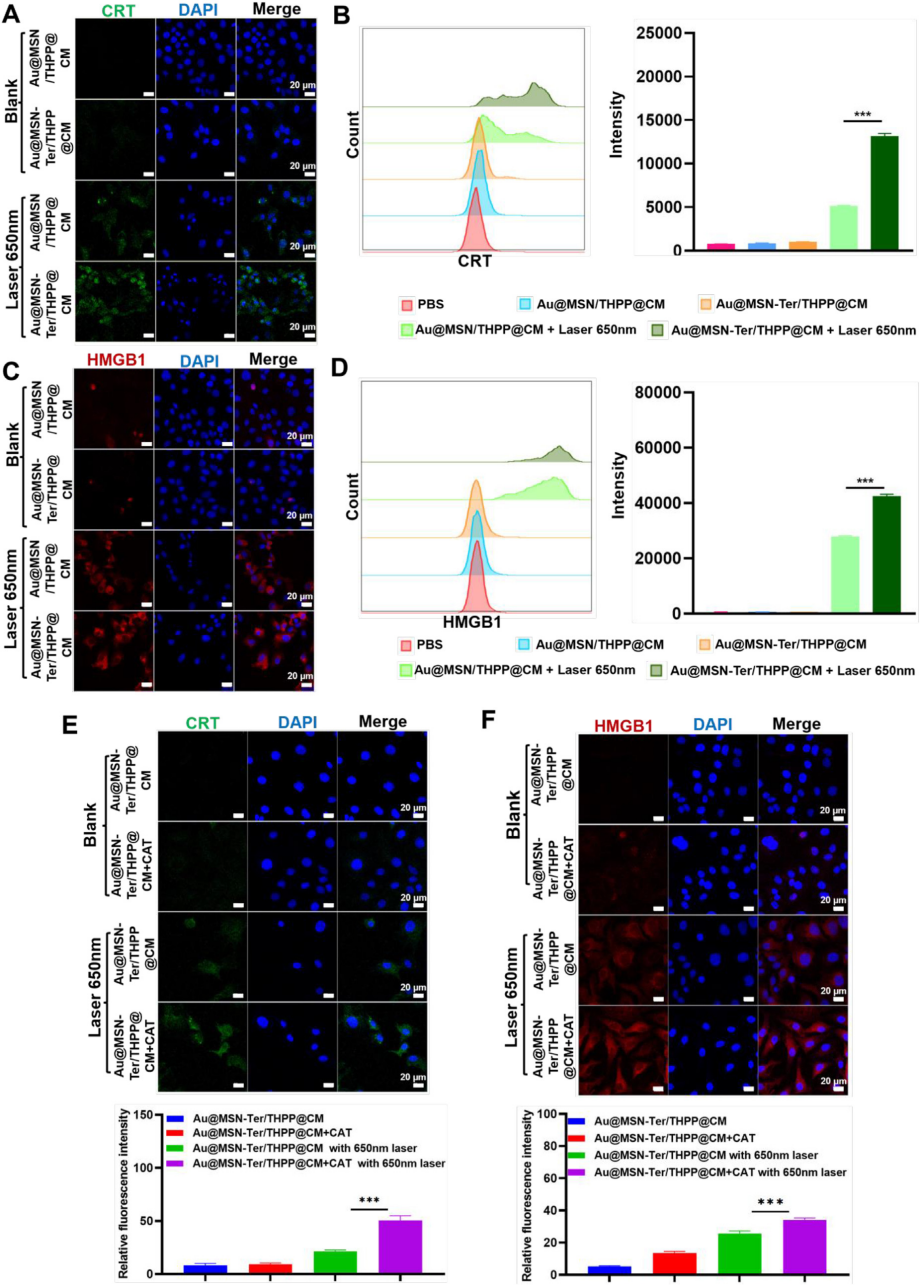
Confocal microscope images of CRT and HMGB1 stained SKOV3 cells after Au@MSN/THPP@CM or Au@MSN-Ter/THPP@CM NPs treatment. Cells were stained with CRT (A) and HMGB1 (C) and imaged with confocal microscope after 24 ​h of Au@MSN/THPP@CM or Au@MSN-Ter/THPP@CM NPs co-incubation and following 650 ​nm laser (0.4 ​W/cm^2^, 5 ​min) irradiation. Flow cytometry analysis and quantifications of CRT (B) or HMGB1 (D) positive cells after 24 ​h of Au@MSN/THPP@CM or Au@MSN-Ter/THPP@CM NPs co-incubation and following 650 ​nm laser (0.4 ​W/cm^2^, 5 ​min) irradiation. Images of CRT (E) and HMGB1 (F) stained SKOV3 cells after treated with Au@MSN-Ter/THPP@CM NPs and selectively treated with free CAT and 650 ​nm laser irradiation (0.4 ​W/cm^2^, 5 ​min). (Green: CRT; Red: HMGB1; Blue: DAPI; Scale bar: 20 ​μm). (For interpretation of the references to color in this figure legend, the reader is referred to the Web version of this article.)

We have shown that the Au@MSN-Ter/THPP@CM NPs can possibly induce photodynamic reaction cell death. Therefore, we further tested the ICD level by analyzing of two typical biomarkers Calreticulin (CRT) and high mobility group box 1 (HMGB1). The CRT protein is mainly expressed in ER, while under stress, the CRT is transferred on to the surface of cell membrane and exposed as an “eat me” signal for immune system. And for HMGB1, as an activator for immune system, it is mainly located in the cell nucleus under normal condition, but it is released into extracellular matrix in stress condition [[34], [35], [36]]. After SKOV3 cells were treated with different NPs, the cells were separately stained with FITC labeled CRT and HMGB1 primary antibodies. Therefore, the fluorescent signal of FITC can be used to monitor the protein level and location change of CRT and HMGB1. And to better distinguish the CRT and HMGB1, we changed the green color of FITC-HMGB1 fluorescent signal to red. As shown in Fig. 3 A and C, the green CRT signal and red HMGB1 signal increased obviously in Au@MSN/THPP@CM or Au@MSN-Ter/THPP@CM NPs with 650 ​nm laser group, the increase was especially significant in Au@MSN-Ter/THPP@CM NPs with 650 ​nm laser group. Quantitative experiments with flow cytometry also revealed the same conclusion, under the irradiation of 650 ​nm laser, the fluorescence intensity of CRT and HMGB1 of cells treated with Au@MSN/THPP@CM NPs increased, while Au@MSN Ter/THPP@CM NPs group showed the highest fluorescence intensity. When no laser was present, the fluorescence intensity was almost unchanged compared with the control group. (Fig. 3 B, D). These results indicated that Au@MSN/THPP@CM or Au@MSN-Ter/THPP@CM NPs treatment with 650 ​nm laser can significantly stimulate the CRT and HMGB1 expression. In addition, the ER targeting ligand Ter modified Au@MSN-Ter/THPP@CM NPs had better ICD provoking ability than Au@MSN/THPP@CM NPs, indicating the importance of using ER targeting.

As we have designed, we were expected to utilize the CAT, which can catalyze H_2_O_2_ to produce O_2_ to enhance Au@MSN-Ter/THPP@CM NPs induced photodynamic reactions in the tumor cells. Therefore, we treated the SKOV3 cells with 5 ​mg/mL CAT and 10 ​mg/mL Au@MSN-Ter/THPP@CM NPs, the concentration of which were shown to be the maximum dose that would not lead to significant cell death in SKOV3 cells (Supplementary Fig. 11), and the cells were treated with 650 ​nm laser irradiation. The result in Fig. 3 E and F shown that, the green CRT signal and red HMGB1 signal were only slightly increased Au@MSN-Ter/THPP@CM with 650 ​nm laser or Au@MSN-Ter/THPP@CM ​+ ​CAT treated SKOV3 cells. And the green CRT signal and red HMGB1 signal had higher intensity in 650 ​nm laser and Au@MSN-Ter/THPP@CM ​+ ​CAT treated SKOV3 cells. All those results indicated that the Au@MSN-Ter/THPP@CM NPs and CAT treatment can induce enhanced ICD activation under 650 ​nm laser irradiation.

### 3.4 Au@MSN-Ter/THPP@CM@GelMA and Au@MSN-Ter/THPP@CM@GelMA/CAT biomimetic nano@microgel preparation and characterization

In recent years, gelatin methacryloyl (GelMA) hydrogels have received more and more attention in the applications of drug delivery, tissue engineering and some other medical purposes owing to their excellent biocompatibility and biodegradability [17,30,37]. For example, the GelMA microspheres formed by crosslinked gelatin molecules under UV light is an ideal drug or cell carrier that can be used for cancer therapy by in situ injection and also site-specific cell transplantation [[38], [39], [40]]. Thereby, we further loaded the Au@MSN-Ter/THPP@CM NPs together with CAT into GelMA microsphere to form the Au@MSN-Ter/THPP@CM@GelMA/CAT biomimetic nano@microgel. We expect that the prepared biomimetic nano@microgel can be used for Ovarian cancer treatment through enhanced photodynamic reactions and photothermal combined therapy by in-situ injection. As shown in the Schematic Figure, the biomimetic nano@microgel Au@MSN-Ter/THPP@CM@GelMA/CAT was prepared on a microfluidic chip. Briefly, CAT (5 ​mg/mL), photocrosslinker (10 ​mg/mL), gelatin methacrylate (150 ​mg/mL) and Au@MSN-Ter/THPP@CM NPs (10 ​mg/mL) were firstly dispersed in PBS solution as an internal flow. Next, the mixture was flow focused by the external flow (mineral oil with 5% Span-80) to form droplet, and then cured by UV irradiation. Finally, the biomimetic nano@microgel Au@MSN-Ter/THPP@CM@GelMA/CAT was collected by centrifugation. In order to maximize the carrying capacity of the biomimetic nano@microgel, we have tried to prepare the Au@MSN-Ter/THPP@CM@GelMA/CAT microspheres with different Au@MSN-Ter/THPP@CM NPs concentrations (0, 5, 10 and 15 ​mg/mL). We found that 10 ​mg/mL was the highest Au@MSN-Ter/THPP@CM NPs’ concentration that could be used for the preparation of Au@MSN-Ter/THPP@CM@GelMA/CAT microspheres, because the 15 ​mg/mL was too high NPs' concentration that destroyed the basic skeleton of the microgel and made it impossible to solidify. Therefore 10 ​mg/mL of Au@MSN-Ter/THPP@CM NP was used for the final biomimetic nano@microgel preparation. And based on cell cytotoxicity study of CAT in SKOV3 cells, we selected 5 ​mg/mL CAT, which was the highest concentration that did not induce considerable cell death in the test (Supplementary Fig. 11).

**Fig. 4:**
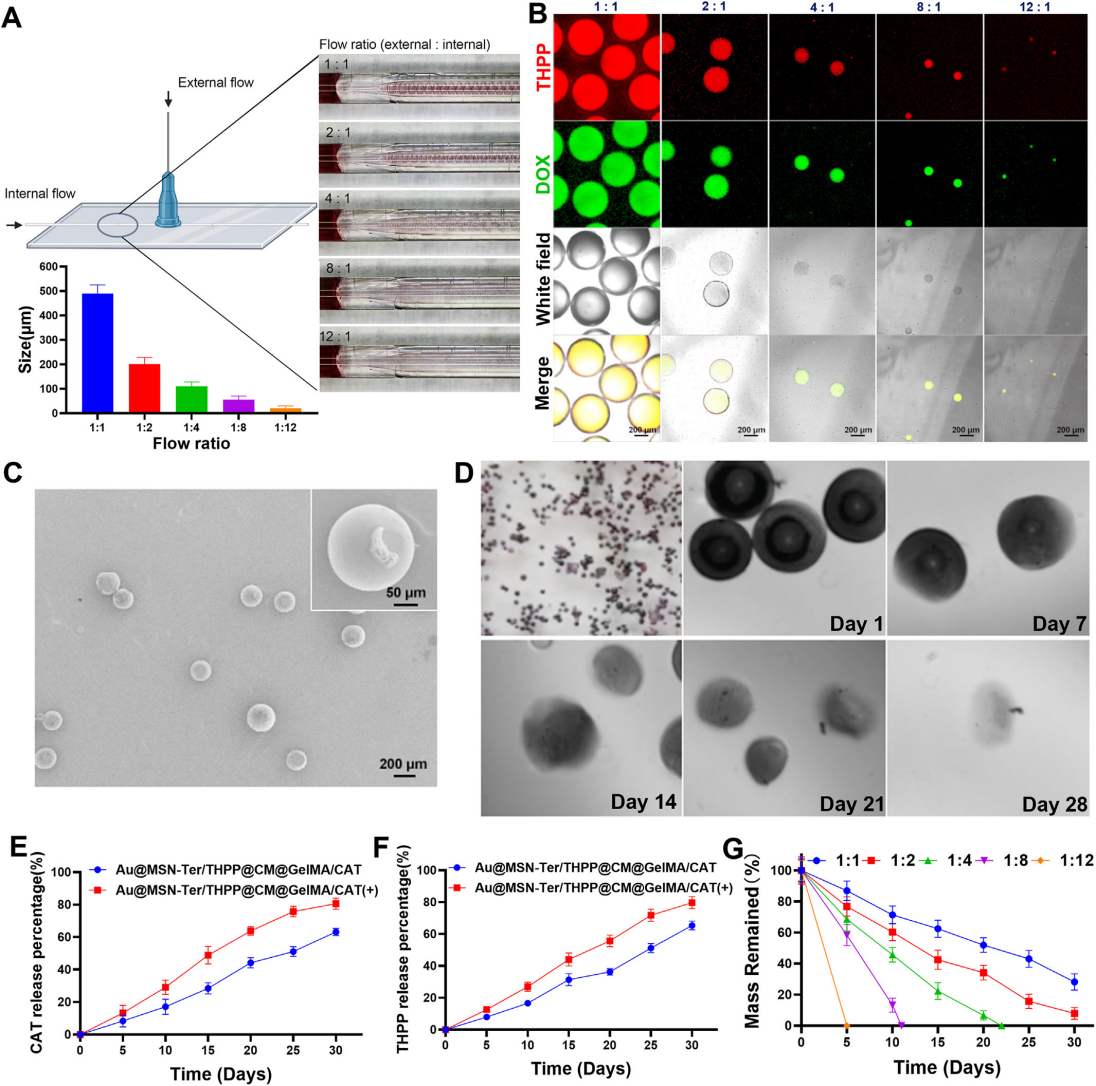
Morphology and CAT/THPP releasing of Au@MSN-Ter/THPP@CM@GelMA/CAT microsphere. Illustration, particle size (A) and confocal microscope images (B) of Au@MSN-Ter/THPP@CM@GelMA/CAT microspheres with indicated external (5% Span 80 in mineral oil) and internal (mixture of NPs, DOX and GELMA) flow rate; C. SEM images of Au@MSN-Ter/THPP@CM@GelMA/CAT microsphere formed by external and internal flow rate of 2:1; D. Microscope images of Au@MSN-Ter/THPP@CM@GelMA/CAT microspheres at different time point; CAT (E) and THPP (F) releasing from Au@MSN-Ter/THPP@CM@GelMA/CAT microspheres in PBS buffer with or without laser (980 ​nm, 1 ​W/cm^2^, 10 ​min); G. The degradation curve of different Au@MSN-Ter/THPP@CM@GelMA/CAT microspheres formed by different flow ratio.

Compared with mechanical emulsification method, the microfluidic method that we used can produce highly monodispersed microgels and effectively avoid the leakage of NPs even under mechanical stirring or ultrasonication. The encapsulation efficiency of Au@MSN-Ter/THPP@CM NPs and CAT by Au@MSN-Ter/THPP@CM@GelMA/CAT microspheres was 89.1% and 91.5%. In addition, by controlling the flow rate of the internal and external channels, Au@MSN-Ter/THPP@CM@GelMA/CAT microspheres of different sizes could be easily prepared. As the results shown in Fig. 4 A, we first fixed the external flow (5% Span 80 in mineral oil) speed at 3 ​mL/h and change the internal flow (mixture of NPs, CAT and GelMA) speed, the diameter of Au@MSN-Ter/THPP@CM@GelMA/CAT microspheres could be produced with the size of 490 ​± ​35, 200 ​± ​28, 110 ​± ​18, 55 ​± ​15 and 20 ​± ​10 ​μm (the corresponding internal: external flow ratio ​= ​1:1, 1:2, 1:4, 1:8, and 1:12). To investigate the composition of the Au@MSN-Ter/THPP@CM@GelMA/CAT microspheres, we utilized a fluorescent drug Doxorubicin (DOX) to mimetic CAT's distribution in Au@MSN-Ter/THPP@CM@GelMA/CAT microspheres. The confocal microscope imaging results showed that the red signal of THPP in Au@MSN-Ter/THPP@CM NPs and green signal represented DOX were evenly dispersed in the microsphere (Fig. 4 B). Next, we further evaluated the stability of Au@MSN-Ter/THPP@CM@GelMA/CAT microspheres by monitoring the degradation of different sizes’ Au@MSN-Ter/THPP@CM@GelMA/CAT biomimetic nano@microgels. The result in Fig. 4 G showed that, the biomimetic nano@microgels were completely degraded at 22 days (internal: external flow ratio of 1:4), 11 days (internal: external flow ratio of 1:8) and 5 days (internal: external flow ratio of 1:12), while there were still 28.2 ​± ​5.2% and 7.9 ​± ​3.8% of Au@MSN-Ter/THPP@CM@GelMA/CAT biomimetic nano@microgels left in flow ratio of 1:1 and 1:2 groups after 22 days even with laser irradiation (980 ​nm laser, 1 ​W/cm^2^, treated for 10 ​min before each time point). This indicated that the degradation of Au@MSN-Ter/THPP@CM@GelMA/CAT microspheres was correlated to its size. In order to reduce the trauma of injection and maximize the sustained release time of the biomimetic nano@microgel, we chose 1:2 as the optimal flow ratio to prepare the final biomimetic nano@microgel (particle size of 200 ​μm). And the microscopy images in Fig. 4 D showed that, the surface damage was detected in nano@microgel after 7 days, and constantly broken and missing was found after 14 and 28 days. In addition, we also evaluated the drying procedure of the GelMA microspheres. Through freeze-drying, we noticed that the water phase nucleated during the freezing process and led to increased surface pores of the GelMA microspheres, which was not good for maintaining the NPs inside and sustained the drug release. To avoid Au@MSN-Ter/THPP@CM NPs losing by the Au@MSN-Ter/THPP@CM@GelMA/CAT microspheres, we applied vacuum drying, and we managed to obtain non-porous GelMA microspheres. As shown in Fig. 4C, the dried Au@MSN-Ter/THPP@CM@GelMA/CAT microspheres had uniform size distribution and very smooth surface under SEM.

Next, we further tested the CAT and NPs' releasing from Au@MSN-Ter/THPP@CM@GelMA/CAT biomimetic nano@microgel. Because THPP can hardly dissolved in water and barely release from the NPs under aqueous condition, we measured the UV absorption of THPP to represent the releasing of NPs and detected the protein concentration of CAT in the releasing buffer by BCA kit to monitor the releasing of CAT. And in order to create a better irradiation condition that can be further used for animal study, we treated the releasing solution with 980 ​nm laser (1 ​W/cm^2^) for 10 ​min every three days. As shown in Fig. 4 E and F, the releasing of THPP and CAT were both in time dependent manner and could last up to 30 days. Furthermore, total THPP releasing was significantly increased from 65.4% ​± ​2.8% to 79.7 ​± ​3.8% (p ​< ​0.001) by 980 ​nm laser irradiation at 30 days' time point, while the CAT releasing increased significantly from 63.2% ​± ​2.2% to 80.6 ​± ​3.4% (p ​< ​0.001) by 980 ​nm laser irradiation at 30 days’ time point. These results demonstrate that the biomimetic nano@microgel Au@MSN-Ter/THPP@CM@GelMA/CAT has good stability and sustained release of NPs.

### 3.5 Evaluating the anti-tumor effect of Au@MSN-Ter/THPP@CM@GelMA/CAT microgel in ovarian cancer cells

**Fig. 5:**
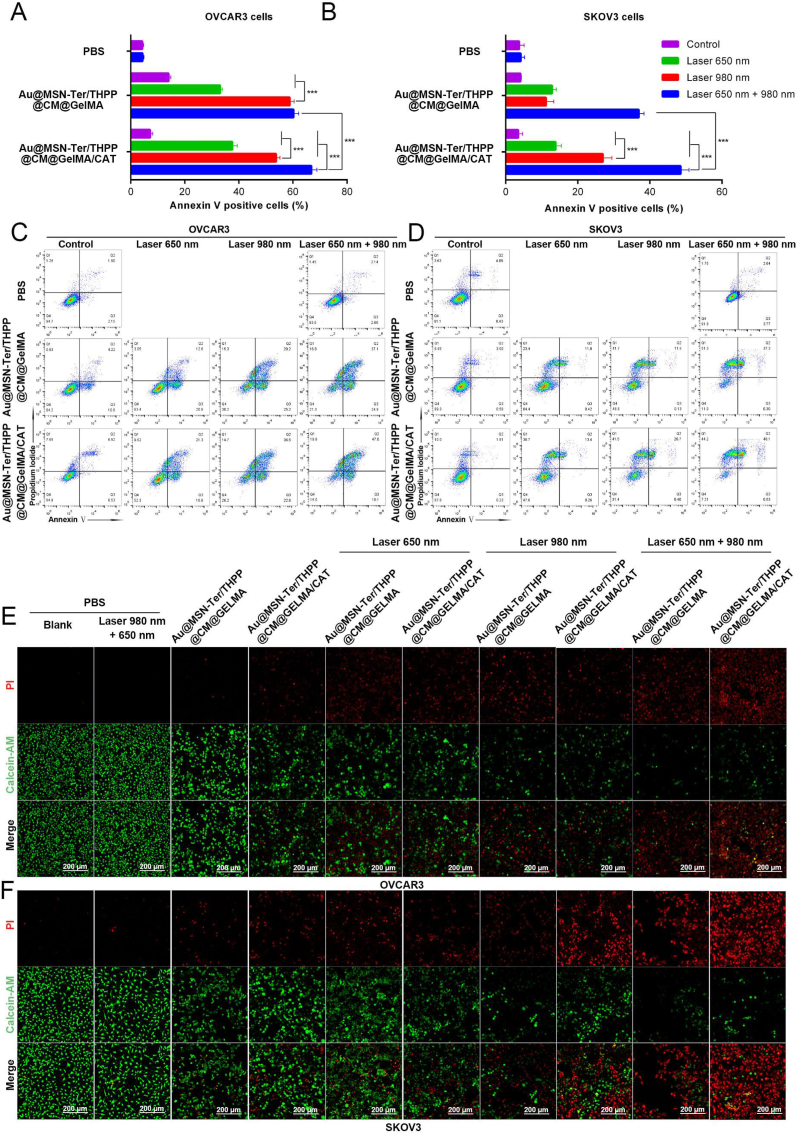
Cytotoxicity of different GelMA microsphere formulations and treatments. A-D. Flow cytometry analysis of apoptotic cells by Annexin V-FITC/PI staining after co-incubation with Au@MSN-Ter/THPP@CM@GelMA or Au@MSN-Ter/THPP@CM@GelMA/CAT for 48 ​h and following 650 ​nm laser (0.4 ​W/cm^2^, 5 ​min) and/or 980 ​nm laser (1 ​W/cm^2^, 10 ​min) irradiation. E and F. Cell death analysis with confocal microscope after co-incubation with Au@MSN-Ter/THPP@CM@GelMA or Au@MSN-Ter/THPP@CM@GelMA/CAT for 48 ​h and following 650 ​nm laser (0.4 ​W/cm^2^, 5 ​min) and/or 980 ​nm laser (1 ​W/cm^2^, 10 ​min) irradiation (Red: PI; Green: Calcein-AM; Scale bar: 200 ​μm). (For interpretation of the references to color in this figure legend, the reader is referred to the Web version of this article.)

We have proofed that the Au@MSN-Ter/THPP@CM NPs had very good capability to deliver THPP with good biocompatibility, and also evidently showed the cell proliferation inhibition ability of Au@MSN-Ter/THPP@CM NPs under both 650 ​nm laser and 980 ​nm laser irradiation. In order to further evaluate the anti-tumor effect of the Au@MSN-Ter/THPP@CM@GelMA/CAT microgel, we performed live-dead cell staining and Annexin Ⅴ, PI double staining assays to test the effect of Au@MSN-Ter/THPP@CM@GelMA/CAT on cell survival in SKOV3 and OVCAR3 cells. The results in Fig. 5 showed that, the Annexin Ⅴ positive cells were distinctly increased by either Au@MSN-Ter/THPP@CM@GelMA or Au@MSN-Ter/THPP@CM@GelMA/CAT treatment for 48 ​h, regardless of single laser (650 ​nm laser or 980 ​nm laser) treatment or double lasers treatment in both two cell lines (Fig. 5 A, B, C and D). While compared to single laser treated groups, Au@MSN-Ter/THPP@CM@GelMA/CAT together with both two lasers treated groups showed more significantly increased Annexin Ⅴ positive cell populations in both SKOV3 and OVCAR3 cells. Most importantly, the Annexin Ⅴ positive cell populations in Au@MSN-Ter/THPP@CM@GelMA/CAT and two lasers treated groups were more than Au@MSN-Ter/THPP@CM@GelMA and two lasers without Au@MSN-Ter/THPP@CM@GelMA treated groups in both SKOV3 and OVCAR3 cells. Because Annexin V is a membrane protein that can only binding to the damaged cell membrane, thus the fluorescent signal of FITC labeled Annexin V were commonly used to detect the apoptotic cells. Therefore, our results demonstrated that the treatment of Au@MSN-Ter/THPP@CM@GelMA or Au@MSN-Ter/THPP@CM@GelMA/CAT can significantly lead to both SKOV3 and OVCAR3 cells apoptosis. And the additional laser irradiation (650 ​nm laser or 980 ​nm laser), especially the two lasers treatment was a considerable promotion factor for cell apoptosis.

Since the red fluorescent nuclei dye PI can only get in to the cells with damaged membrane, while the Calcein-AM can be catalyzed in to green fluorescent Calcein and retained only in living cells. Therefore, the double staining of PI and Calcein-AM were always used to distinguish the living and dead cells. As the live-dead cell staining results in Fig. 5 E and F shown, the red PI fluorescent signal was significantly increased while the green Calcein-AM fluorescent signal was decreased in all Au@MSN-Ter/THPP@CM@GelMA or Au@MSN-Ter/THPP@CM@GelMA/CAT treated SKOV3 and OVCAR3 cells, and the additional laser treatments (either 650 ​nm laser and/or 980 ​nm laser) came to be an acceleration factor for the tendency. While in all Au@MSN-Ter/THPP@CM@GelMA or Au@MSN-Ter/THPP@CM@GelMA/CAT treated SKOV3 and OVCAR3 cells, compare to single laser (650 ​nm laser or 980 ​nm laser) treated groups, double lasers (650 ​nm laser and 980 ​nm laser) treatments were more significant in increasing the red PI fluorescent signal and decreasing the green Calcein-AM fluorescent signal. Moreover, the increasing of red PI fluorescent signal and decreasing of green Calcein-AM fluorescent signal in Au@MSN-Ter/THPP@CM@GelMA/CAT with double lasers treated groups were more obviously than Au@MSN-Ter/THPP@CM@GelMA with double lasers in both SKOV3 and OVCAR3 cells. All of these results demonstrated that both the formulations of Au@MSN-Ter/THPP@CM@GelMA and Au@MSN-Ter/THPP@CM@GelMA/CAT together with laser 650 ​nm and/or 980 ​nm treatment can lead to significant cell death and apoptosis in both OVCAR3 and SKOV3 cells. And compared to Au@MSN-Ter/THPP@CM@GelMA, Au@MSN-Ter/THPP@CM@GelMA/CAT is more efficient for inducing the cell death and apoptosis.

### 3.6 Evaluate the ability of ICD activation by Au@MSN-Ter/THPP@CM@GelMA/CAT in ovarian cancer cells

**Fig. 6:**
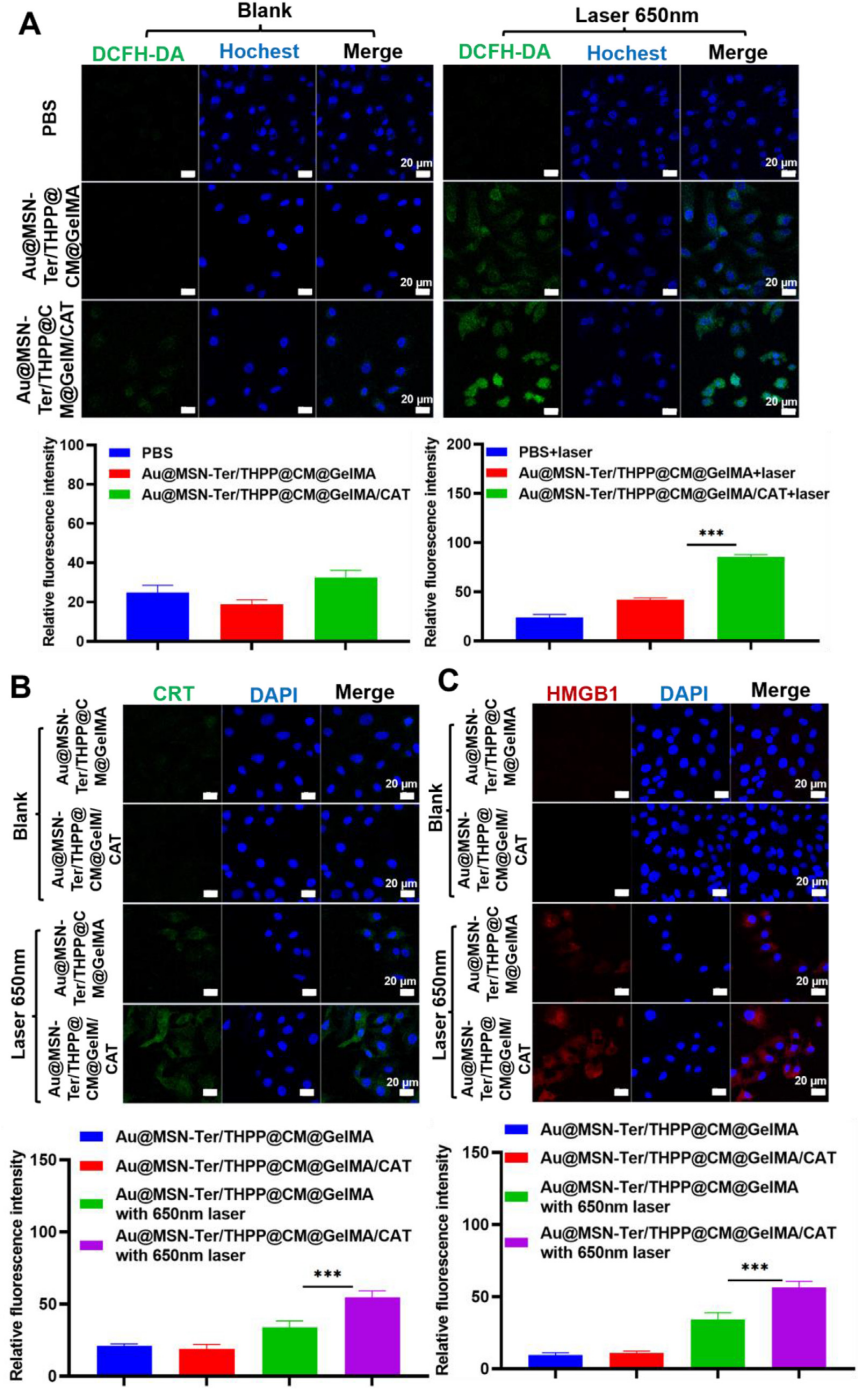
Confocal microscope images of SKOV3 cell after co-incubation with Au@MSN-Ter/THPP@CM@GelMA or Au@MSN-Ter/THPP@CM@GelMA/CAT for 48 ​h and selectively treated with 650 ​nm laser (0.4 ​W/cm^2^) for 5 ​min. A. Images of DCFH-DA stained SKOV3 cells and quantifications (Green: DCFH-DA; Blue: Hochest; Scale bar: 20 ​μm); Images of CRT (B) and HMGB1 (C) primary antibodies stained SKOV3 cells and quantifications (Green: CRT; Red: HMGB1; Blue: DAPI; Scale bar: 20 ​μm). (For interpretation of the references to color in this figure legend, the reader is referred to the Web version of this article.)

To further confirm the Au@MSN-Ter/THPP@CM@GelMA/CAT generated enhanced photodynamic reactions design, we detected the ROS level by a ROS indicator DCFH-DA in Au@MSN-Ter/THPP@CM@GelMA and Au@MSN-Ter/THPP@CM@GelMA/CAT treated SKOV3 cells. The DCFH-DA is a non-fluorescent molecule, which can be catalyzed and oxidated in to a strong fluorescent product DCF, thus can be used to analysis the ROS level of cells. As the results showed in Fig. 6, weak green fluorescent signal of DCF could be only observed in Au@MSN-Ter/THPP@CM@GelMA/CAT treated SKOV3 cells after 48 ​h. While in either Au@MSN-Ter/THPP@CM@GelMA or Au@MSN-Ter/THPP@CM@GelMA/CAT treated SKOV3 cells, the green fluorescent signal were dramatically increased by extra laser 650 ​nm treatment, especially in Au@MSN-Ter/THPP@CM@GelMA/CAT group (Fig. 6 A). The results evidently showed that the ROS level in either Au@MSN-Ter/THPP@CM@GelMA or Au@MSN-Ter/THPP@CM@GelMA/CAT treated SKOV3 cells were significantly increased by laser 650 ​nm. Therefore, we further evaluated the consequence induced by high level of ROS in SKOV3 cells.

Prolonged ER stress, which can be induced by high level of ROS is one of the leading causes for ICD triggered anti-tumor immune response [41,42]. As shown in our design (Schematic figure), we modified the ER targeting ligand Ter on the surface of Au@MSN-Ter/THPP NPs. The Au@MSN-Ter/THPP NPs were successfully conducted to the ER once they escaped from the endo/lysosome (Supplementary Fig. 6). We believed that high level of ROS produced by the accumulated Au@MSN-Ter/THPP NPs in ER could certainly lead to irreversible ER stress under laser 650 ​nm irradiation. Therefore, we detected two key biomarkers of ICD to evaluate the potential ability of anti-tumor immune response provoking by Au@MSN-Ter/THPP@CM@GelMA/CAT. As shown in Fig. 6 B, C, both the fluorescent signals of CRT and HMGB1 on the cell membrane of SKOV3 cells were dramatically increased in either Au@MSN-Ter/THPP@CM@GelMA or Au@MSN-Ter/THPP@CM@GelMA/CAT treated groups with additional laser 650 ​nm treatment, and most obviously detected in Au@MSN-Ter/THPP@CM@GelMA/CAT plus laser 650 ​nm group. The increasing of both CRT and HMGB1 fluorescent signals on the cell membrane of SKOV3 cells sufficiently indicated that the ICD status were in extremely high level in Au@MSN-Ter/THPP@CM@GelMA/CAT together with laser 650 ​nm treated SKOV3 cells.

### 3.7 Biodistribution and anti-tumor effects in ovarian cancer orthotopic implantation model

**Fig. 7:**
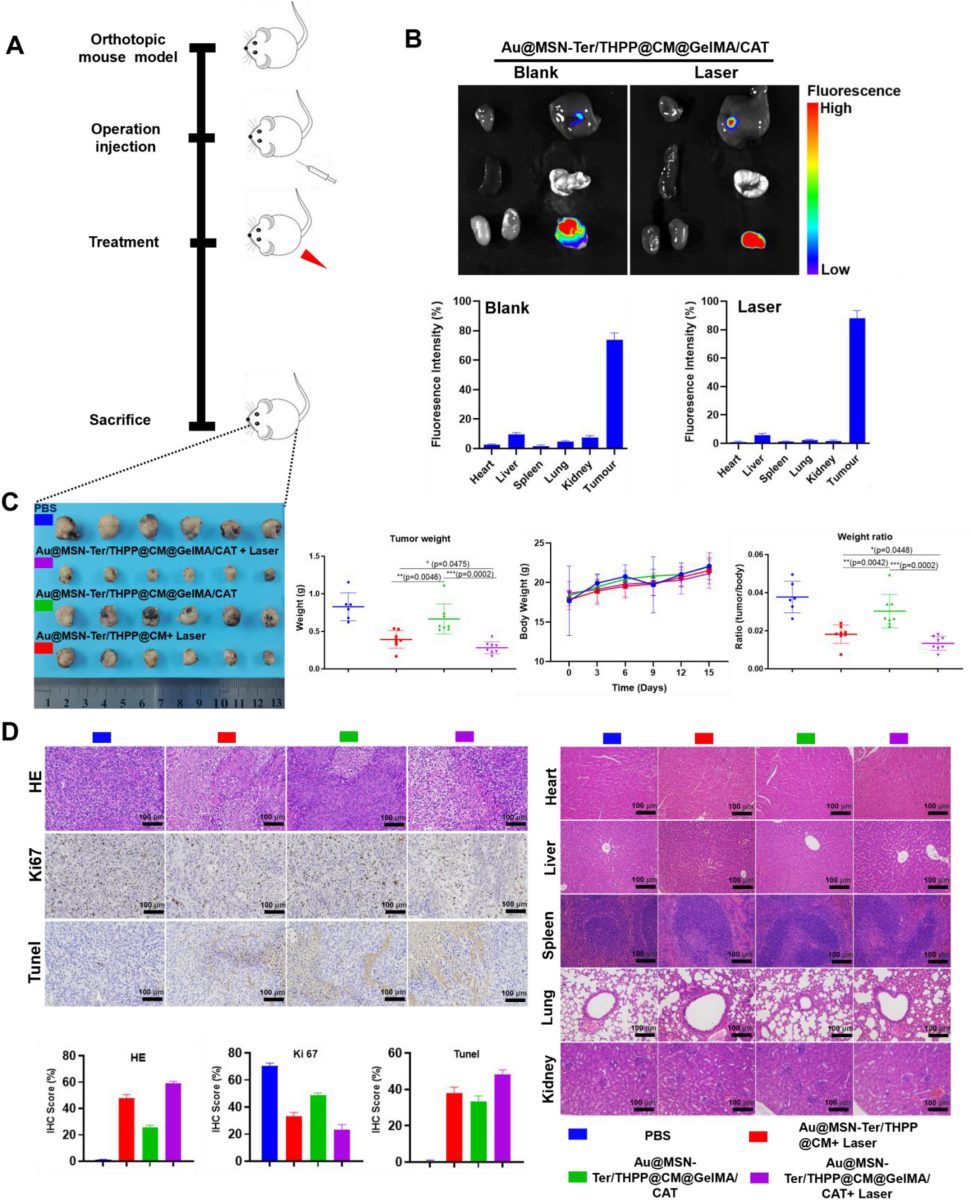
Bio-imaging, tumors growth and histological analysis after different formulations of peritumoral administration. A. Illustration of animal experiment. B. Organ tissues THPP fluorescent signal captured by in vivo imaging system and quantifications (From left to right and up to down panels: Heart, liver, spleen, lung, kidney, and tumor). C. Data of tumor images, tumor weight, mice body weight and weight ratio (tumor/body) (n ​≥ ​6). D. HE, Ki67 and Tunel staining images and quantifications of tumor, and HE staining images of different organs (n ​≥ ​6, 20 ​× ​, scale bar: 100 ​μm).

All of our previous results showed that the Au@MSN-Ter/THPP@CM@GelMA/CAT was with sustained NPs releasing and enhanced anti-tumor activity in Ovarian cancer cells synergistically with 650 ​nm and 980 ​nm lasers treatment. Moreover, the Au@MSN-Ter/THPP@CM@GelMA/CAT together with 650 ​nm laser treatment had great potential to provoke ICD induced anti-tumor immune response. Therefore, we further performed animal study to evaluate the possible anti-tumor activity of Au@MSN-Ter/THPP@CM@GelMA/CAT in vivo. The experiment was carried out with SKOV3 generated Ovarian cancer orthotopic implantation mice model (Fig. 7 A and Supplementary Fig. 14), and mice were randomly separated into four groups (n ​≥ ​6). PBS treated group was injected with 100 ​μL PBS from tail vain, NPs treated group was injected with 100 ​μL Au@MSN-Ter/THPP@CM NPs in the tumor site, and two another groups were injected with 30 ​μL Au@MSN-Ter/THPP@CM@GelMA/CAT (dose: equal to 2.5 ​mg/kg of THPP) in the tumor site. The PBS, NPs and one Au@MSN-Ter/THPP@CM@GelMA/CAT treated groups were treated lasers (10 ​min of 980 ​nm laser, 5 ​min of 650 ​nm laser) once every three days as scheduled. As shown in Fig. 7 B and Supplementary Fig. 20, after only 6 ​h tail vein injection of Au@MSN-Ter/THPP@CM NPs and followed double lasers treatment, the Au@MSN-Ter/THPP@CM NPs were detected in both liver and cancer tissues. And increased Au@MSN-Ter/THPP@CM NPs accumulation was found in both liver and tumor tissues, as well as in lung and kidney tissues at 24 and 48 ​h. In nano@microgels groups, only few Au@MSN-Ter/THPP@CM NPs were detected in the liver, while large amount of Au@MSN-Ter/THPP@CM NPs were detected in tumor tissues in Au@MSN-Ter/THPP@CM@GelMA/CAT in situ injection groups at 15 days regardless of laser treatment (Fig. 7 B). These results demonstrated that the Au@MSN-Ter/THPP@CM NPs was mainly retention in the tumor tissue after Au@MSN-Ter/THPP@CM@GelMA/CAT microsphere's intratumoral injection. While the tail vein injection of Au@MSN-Ter/THPP@CM NPs also exhibited main organs (such as liver, kidney, and lung) accumulation. Furthermore, the result of tumor weight from sacrificed mice after fifteen days treatment with different formulations and lasers showed that both Au@MSN-Ter/THPP@CM NPs and Au@MSN-Ter/THPP@CM@GelMA/CAT can significantly decrease the tumor weight under double lasers (Fig. 7C). In addition, the tumor weights in either Au@MSN-Ter/THPP@CM NPs or Au@MSN-Ter/THPP@CM@GelMA/CAT together with double lasers treated groups were obviously lower than Au@MSN-Ter/THPP@CM@GelMA/CAT only treated group, and Au@MSN-Ter/THPP@CM@GelMA/CAT together with lasers treated group exhibited the lowest tumor weight result. Subsequently, we sliced the collected tumor and organ tissues and performed HE, Ki 67 and Tunel staining assays to further evaluate the anti-tumor activity and organ toxicity of different formulations. As the result in Fig. 7 D, HE staining showed that compared to PBS treated group, cell death area of tumor tissues were strikingly increased in all different formulations treated groups, especially in Au@MSN-Ter/THPP@CM@GelMA/CAT together with lasers treated group. Correspondingly, the expression of tumor cell proliferation biomarker Ki 67 and apoptotic cells' biomarker Tunel staining were changed in the similar way as the result in HE staining. These results indicated that both formulations and treatments were effectively inhibited the tumor growth, while Au@MSN-Ter/THPP@CM@GelMA/CAT together with lasers was the best selection for the implementation of cancer treatment. And more significantly, both of the formations were non-toxic to the main organs within the used maximum formulation concentrations, because there were no obvious organ tissue damages were observed in the main organs (include heart, liver, spleen, lung and kidney) HE staining results.

## 4 Conclusion and discussion

In this study, we have developed a microfluidic method for fabricating biomimetic Au@MSN-Ter/THPP@CM@GelMA/CAT nano@microgels. These gels consist of cured GelMA microspheres and inner components including CAT and Au@MSN-Ter/THPP@CM NPs. The Au@MSN-Ter/THPP@CM NPs were found to possess excellent photothermal conversion ability and stability. Additionally, the Au@MSN-Ter/THPP@CM@GelMA/CAT nano@microgels demonstrated good biocompatibility and long-term tumor retention capability. Importantly, both in vitro and in vivo studies showed that Au@MSN-Ter/THPP@CM NPs and Au@MSN-Ter/THPP@CM@GelMA/CAT exhibited excellent anti-tumor activity against ovarian cancer when both 650 ​nm and 980 ​nm lasers were applied. These findings provide strong evidence for the potential of these nano@microgels as a promising therapeutic approach for ovarian cancer.

ROS provoking for cancer therapy has always been considered as a good strategy for nano-formulation drugs design [43,44]. The most common ROS include hydrogen peroxides (H_2_O_2_), superoxide (•O_2_^−^), hydroxyl radical (•OH) and singlet oxygen (^1^O_2_). And among them ^1^O_2_ is the most reactive one, which can result in most serious cell damages via reaction with organic molecules that contain double bonds [45]. In this work, we encapsulated CAT together with Au@MSN-Ter/THPP@CM NPs inside of the Au@MSN-Ter/THPP@CM@GelMA/CAT microsphere, the produced O_2_ by released CAT catalyzing H_2_O_2_ served as good source for THPP based photodynamic reaction generated ^1^O_2_ production. As we have proofed in Fig. 6 A, the ROS level was significantly increased in Au@MSN-Ter/THPP@CM@GelMA/CAT and laser 650 ​nm treated SKOV3 cells, and this further induced increasing CRT exposure and HMGB1 releasing (Fig. 6 B and C). Therefore, all of these results evidently proved the enhanced photodynamic reaction design of our Au@MSN-Ter/THPP@CM@GelMA/CAT microgel, and this is a very good strategy of enhanced ROS provoking by photodynamic reaction for cancer therapy.

The photo-thermal response has always been considered as one of the best designs for developing smart nanocarriers [[46], [47], [48]]. However, the results of photothermal therapy (PTT) can be easily affected by various key factors that may decrease the photothermal conversion efficiency, including NP stability, distribution, and the selected light source [49]. In our work, we developed photothermal-responsive Au-MSN NPs as nanocarriers for THPP, thereby combining PTT with PDT. The results, as shown in Fig. 2, Fig. 5, indicate that both cell death and apoptosis were significantly increased in the groups treated with both lasers (650 ​nm and 980 ​nm) compared to those treated with a single laser, whether using Au@MSN-Ter/THPP@CM NPs or Au@MSN-Ter/THPP@CM@GelMA/CAT.

Furthermore, following intratumoral injection, the nano@microgel Au@MSN-Ter/THPP@CM@GelMA/CAT also demonstrated excellent tumor retention ability, as illustrated in Fig. 7 B. This serves as an effective solution to the problem of low accumulation of photosensitizers at the tumor site, which can reduce the amount of photosensitizer used in PDT. Moreover, the results in Fig. 7 show that enhanced anti-tumor activity can be achieved by using Au@MSN-Ter/THPP@CM@GelMA/CAT in conjunction with additional 650 ​nm and 980 ​nm laser irradiation.

In summary, the nano@microgel Au@MSN-Ter/THPP@CM@GelMA/CAT has shown great potential for inducing high levels of ROS and triggering ICD through photothermal and enhanced photodynamic reactions, both in vitro and in vivo. This newly designed biomimetic nano@microgel could be a promising candidate for in situ ovarian cancer therapy.

## Author contributions

**Xiaodong Ma**: Conceptualization, Methodology, Software, Validation Formal analysis, Investigation, Data Curation, Writing - Original Draft, Writing - Review & Editing, Visualization. **Wenhui Zhou**: Methodology, Software, Validation Formal analysis, Investigation, Data Curation, Writing - Original Draft, Writing - Review & Editing, Visualization. **Rong Zhang**: Resources, Funding acquisition. **Canan Zhang**: Resources, Data Curation. **Jiaqi Yan**: Resources, Writing - Original Draft. **Jing Feng**: Resources, Writing - Review & Editing. **Tingyan Shi**: Resources, Funding acquisition. **Jessica M. Rosenholm**: Resources, Writing - Review & Editing. **Xian Shen**: Resources, Writing - Review & Editing. **Hongbo Zhang**: Conceptualization, Methodology, Validation, Resources, Writing - Review & Editing, Visualization, Supervision, Project administration, Funding acquisition.

## Declaration of competing interest

The authors declare that they have no known competing financial interests or personal relationships that could have appeared to influence the work reported in this paper.

## Data Availability

Data will be made available on request.
