# Supplementary material for: Minimally invasive injection of biomimetic Nano@Microgel for in situ ovarian cancer treatment through enhanced photodynamic reactions and photothermal combined therapy
Source: Mater Today Bio. 2023 May 18;20:100663. doi: 10.1016/j.mtbio.2023.100663 (PMC10232889; doi:10.1016/j.mtbio.2023.100663)

**Supplementary Figures**

**Supplementary Figure 1.** Elemental mapping of Au@MSN NPs.


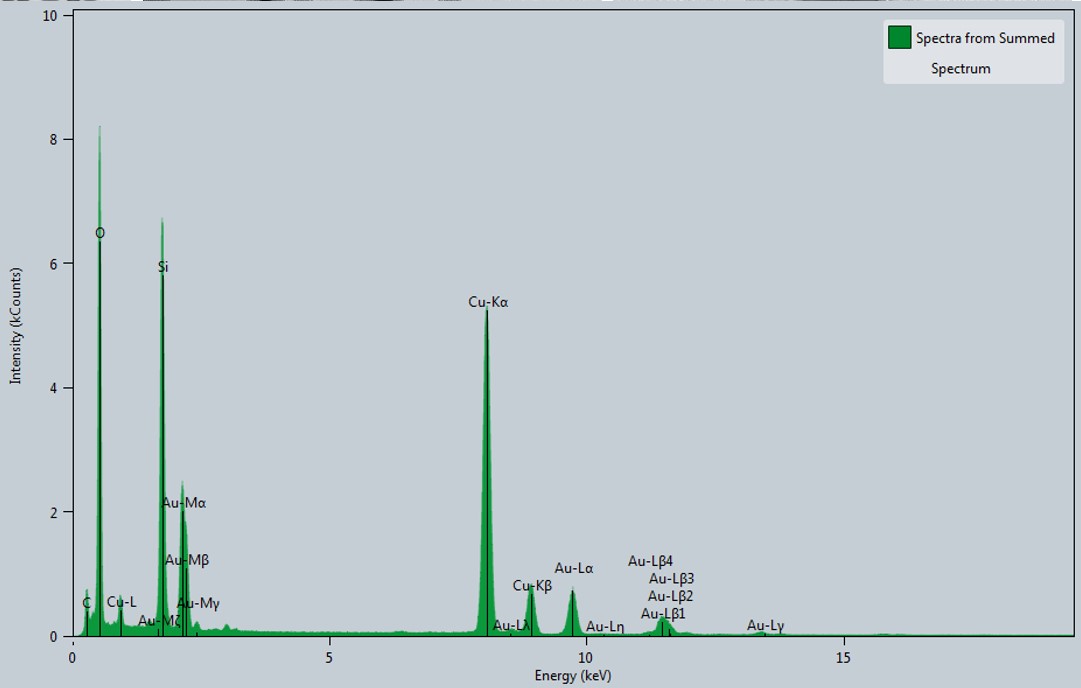


**Supplementary Figure 2.** FTIR analysis of Ter, Au@MSN and Au@MSN-Ter NPs (from up to down panel).


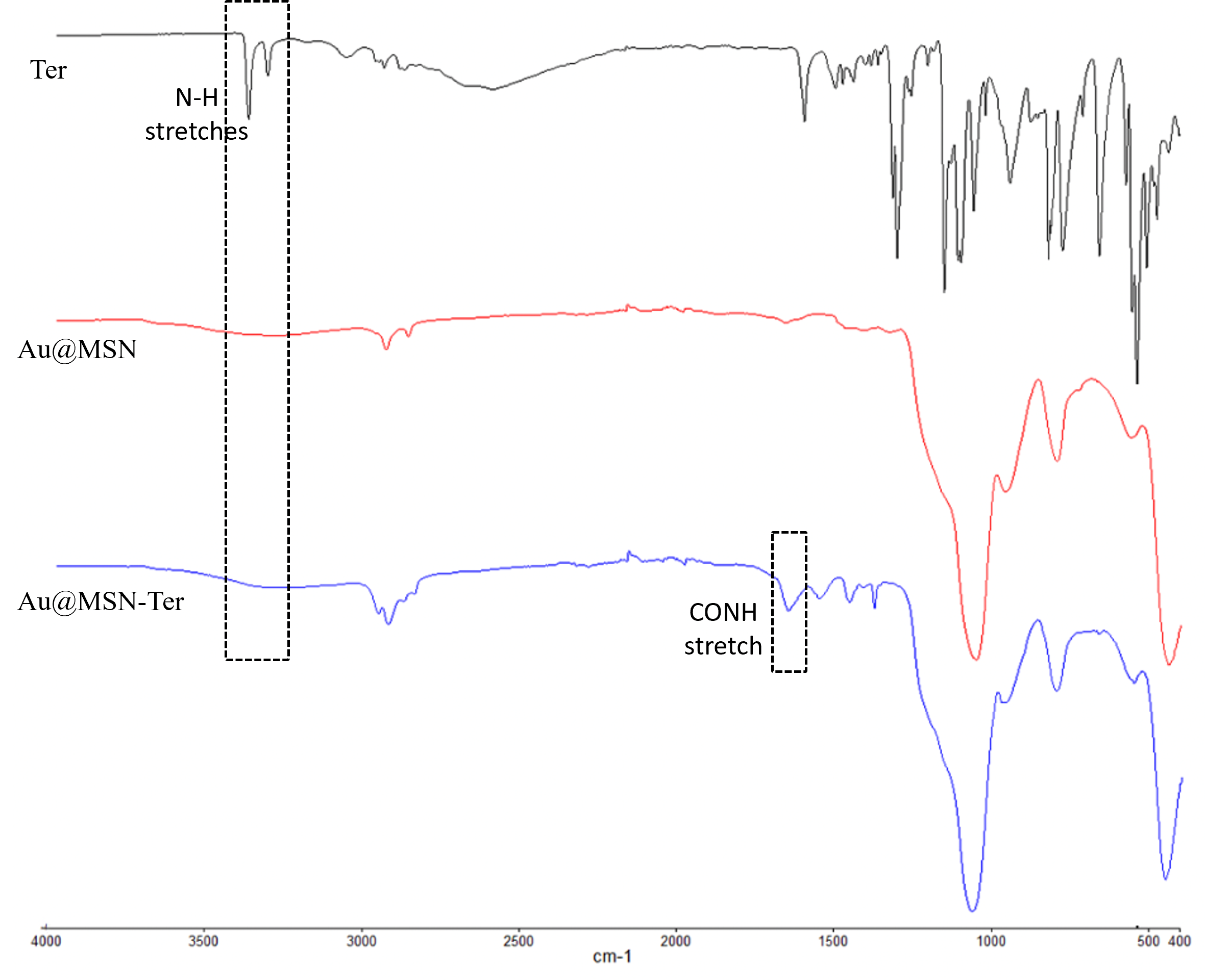


**Supplementary Figure 3.** The stability of Au@MSN-Ter/THPP@CM NPs in culture medium.


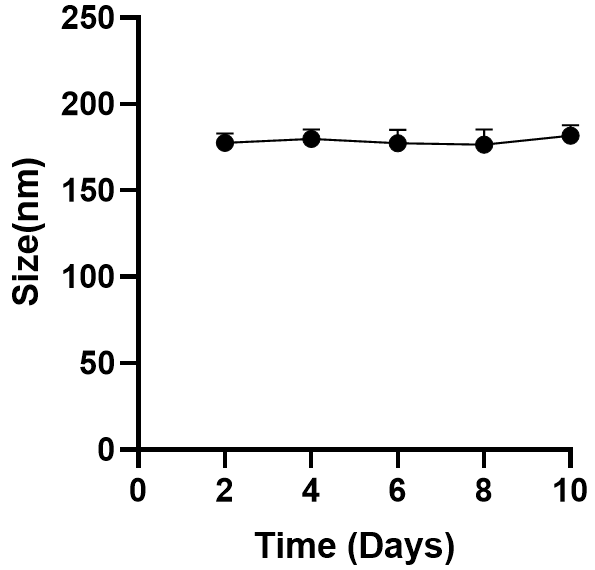

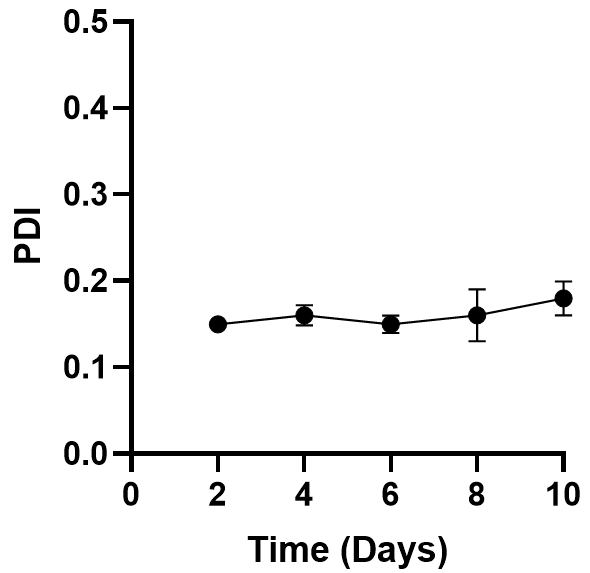


**Supplementary Figure 4.** Time-dependent production of SOSG fluorescence upon light exposure (650 nm, 0.4 W/cm^2^) for THPP and Au@MSN-Ter/THPP@CM NPs.

**
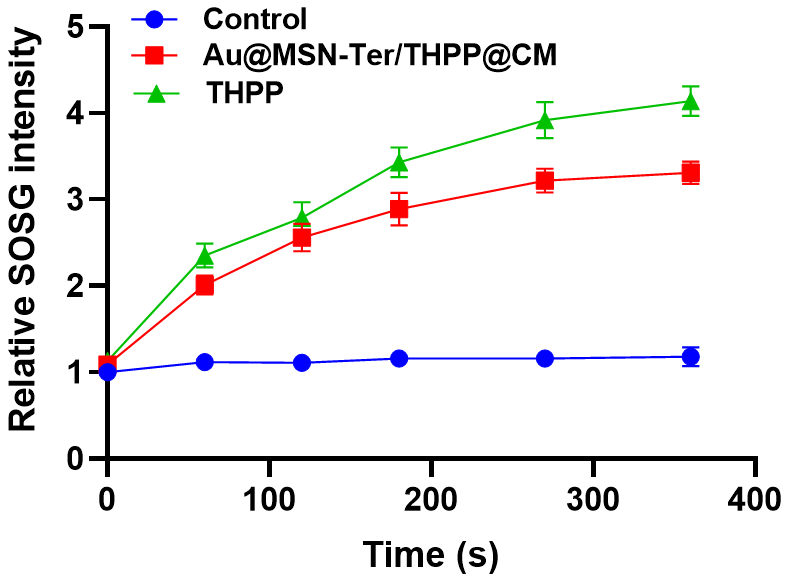
**

**Supplementary Figure 5.** Cell viability of NHDF cells after co-incubation with different NPs for 48 hours and selectively treated with laser irradiation.

**
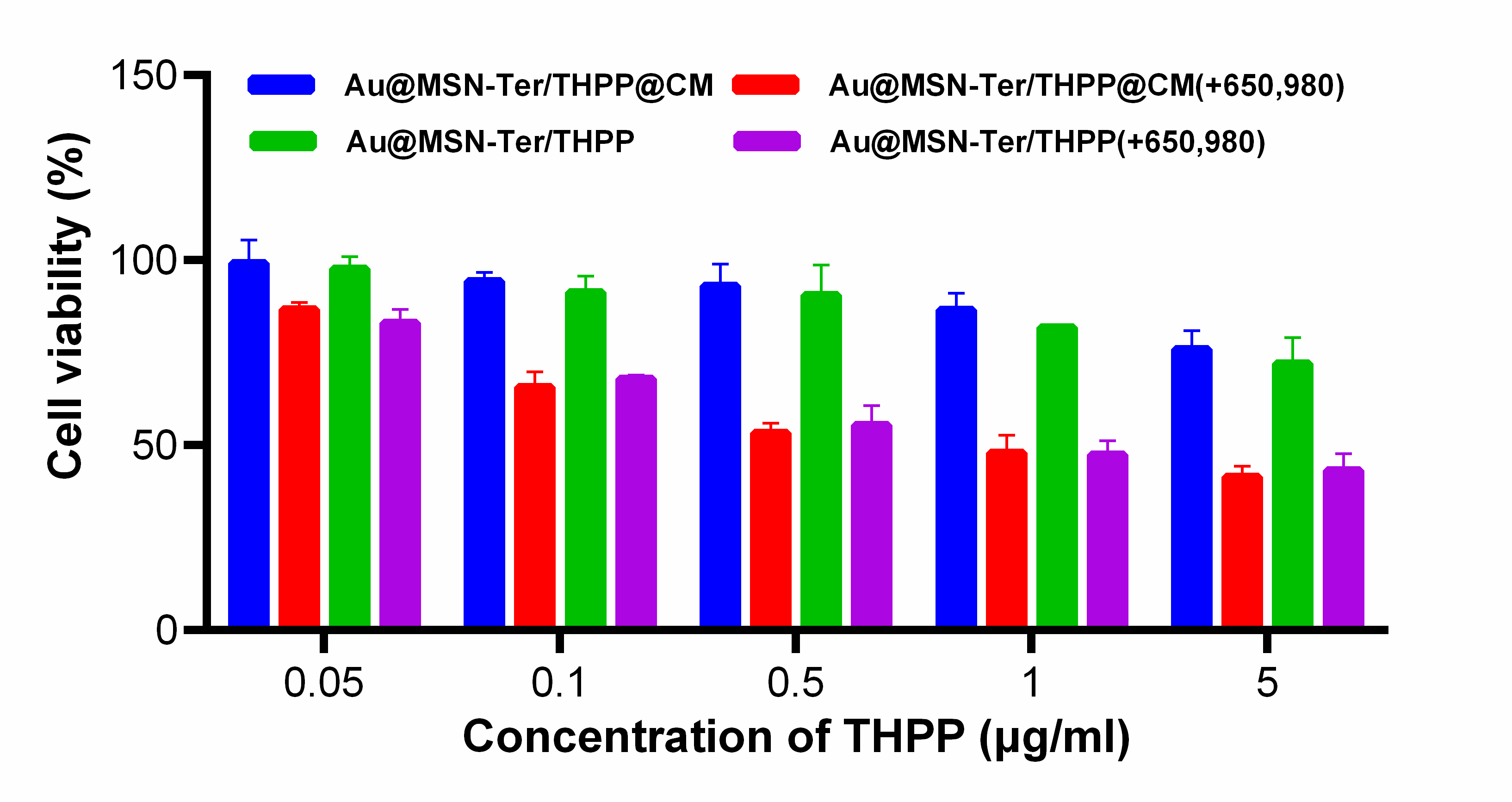
**

**Supplementary Figure 6.** SKOV3 cells were stained with THPP (red), ER-Tracker (green) and imaged with confocal microscope after 24 hours of Au@MSN/THPP@CM or Au@MSN-Ter/THPP@CM NPs co-incubation (Red: THPP; Green: ER-Tracker; Blue: DAPI; Scale bar: 10 μm).


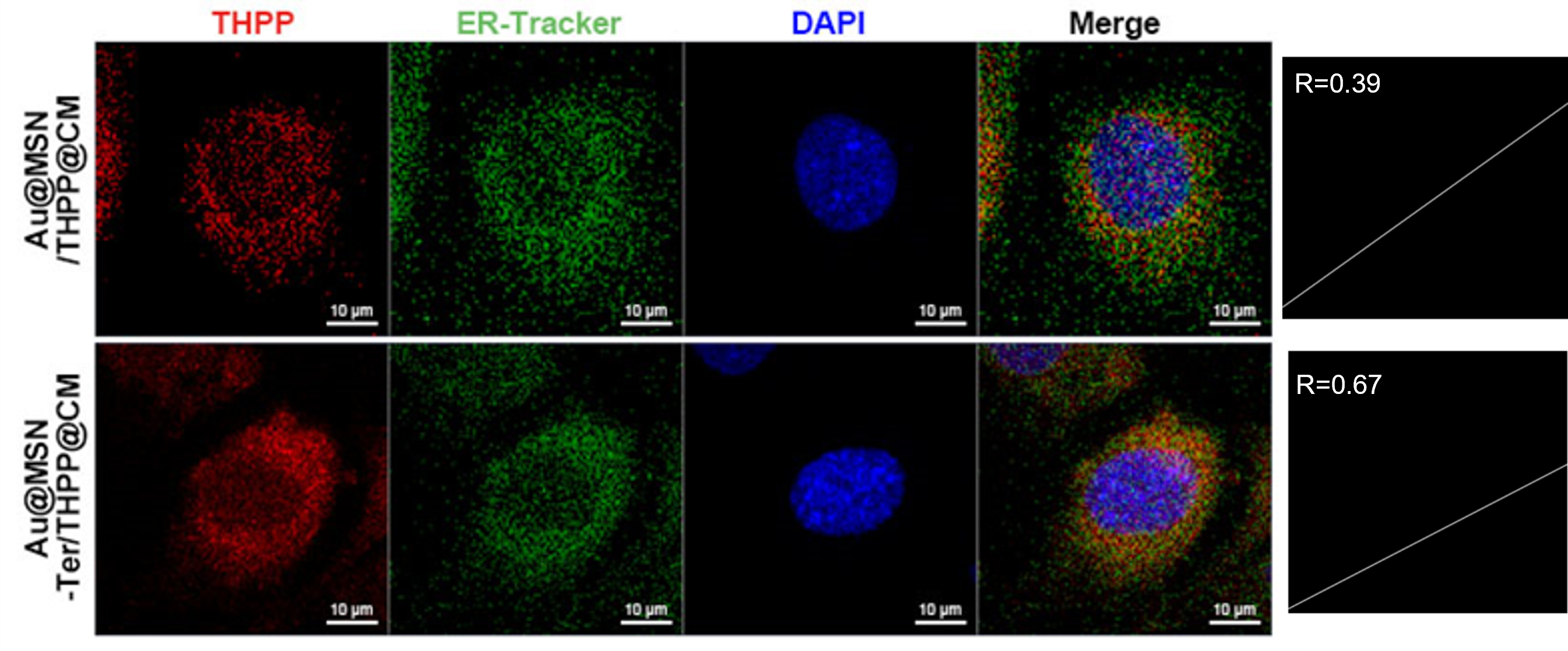


**Supplementary Figure 7.** Confocal microscope imaging (A) and flow cytometry analyzing (B) of THPP positive OVCAR3 cells after co-incubated with Au@MSN-Ter/THPP or Au@MSN-Ter/THPP@CM NPs (Red: THPP; Blue: DAPI; Scale bar: 20 μm). C. Mean fluorescence intensity of THPP in (B) (*** P≤0.001).

**
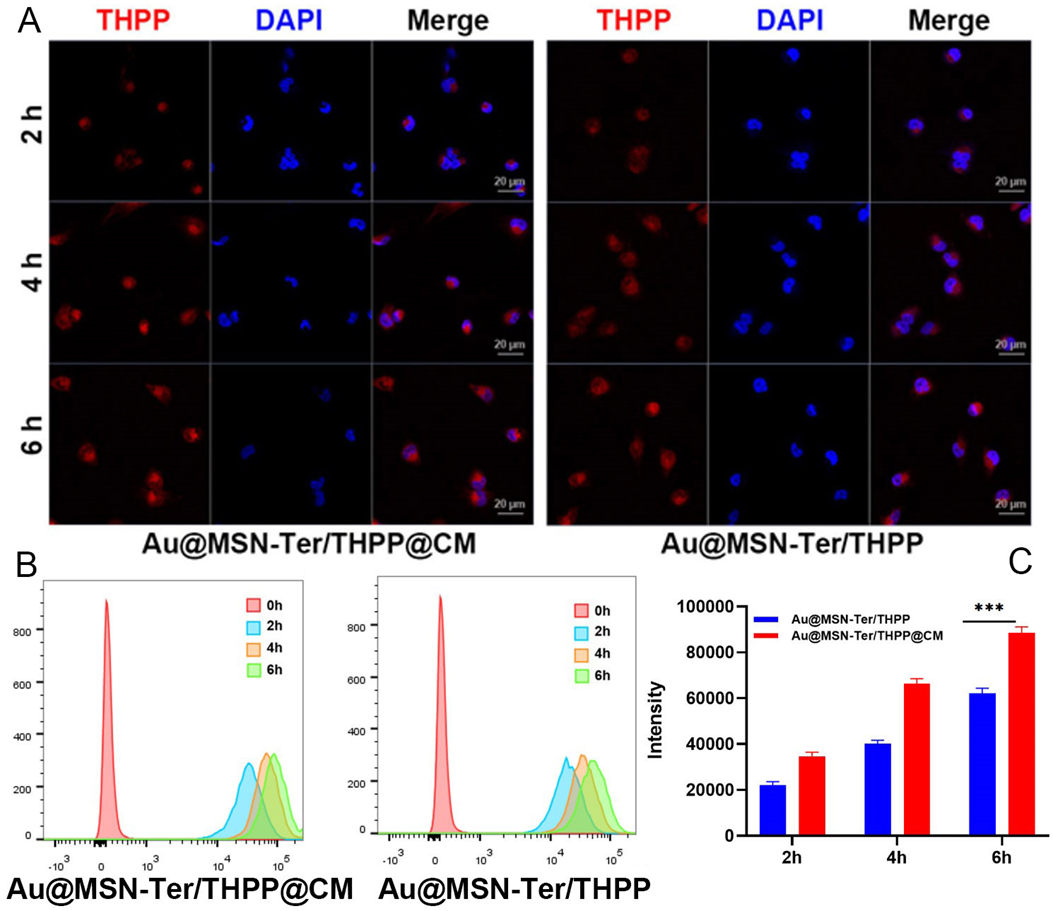
**

**Supplementary Figure 8.** Flow cytometry analyzing of THPP fluorescence signal positive NHDF cells after co-incubation with Au@MSN-Ter/THPP or Au@MSN-Ter/THPP@CM NPs for 48 hours.


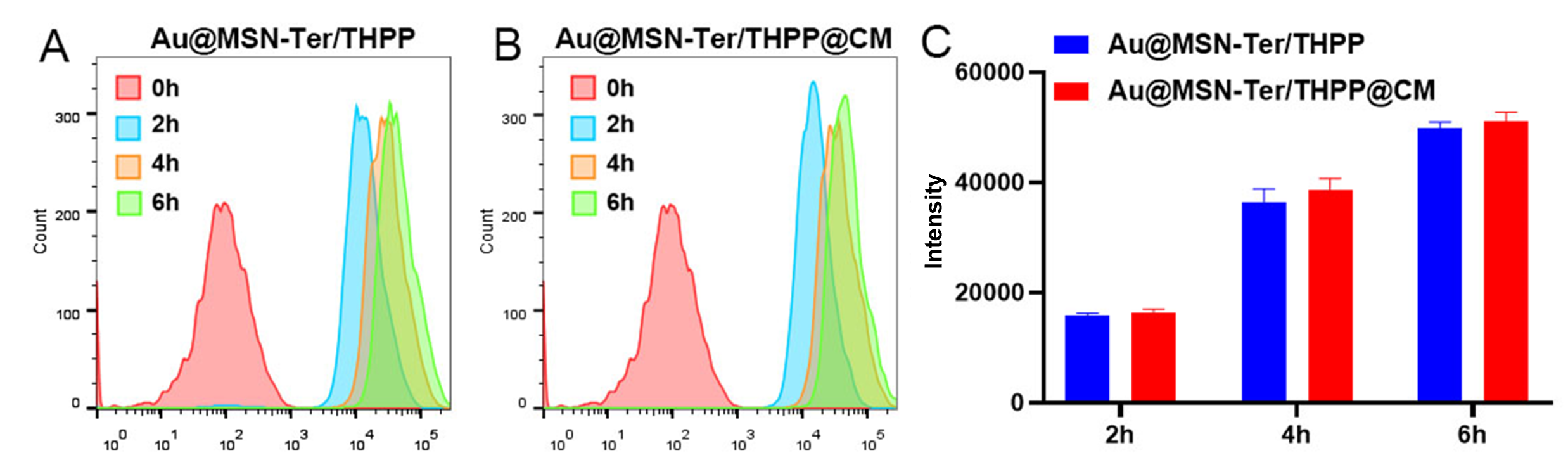


**Supplementary Figure 9.** Confocal images of NHDF cells after co-incubation with Au@MSN-Ter/THPP or Au@MSN-Ter/THPP@CM NPs for 2-6 hours.

**
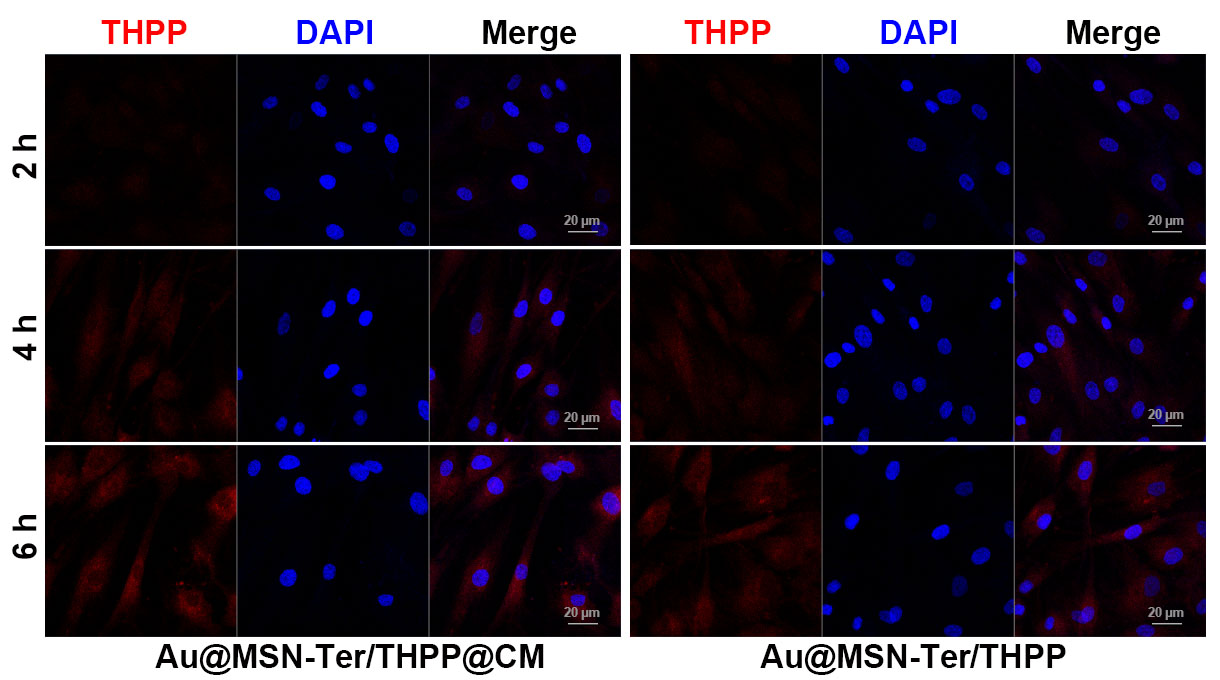
**

**Supplementary Figure 10.** UV–Vis absorption spectra of Au@MSN-Ter NPs.


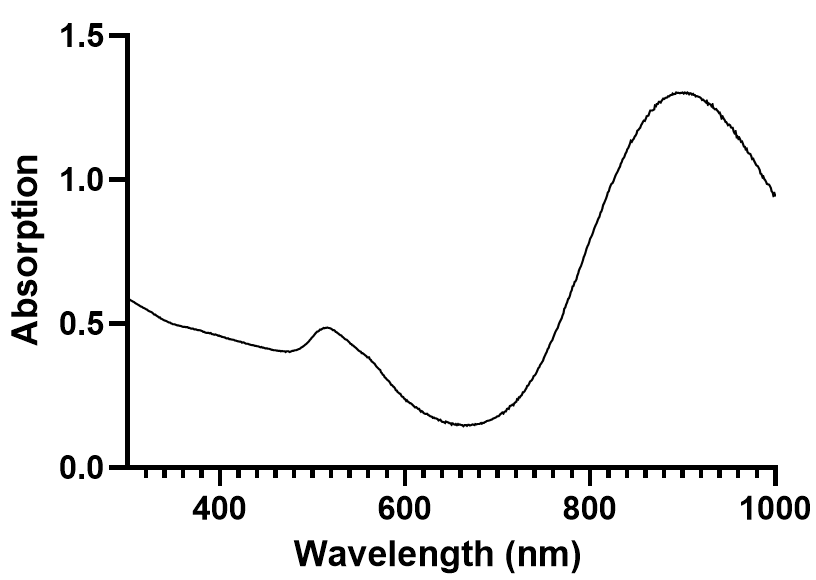


**Supplementary Figure 11.** Cell viability of SKOV3 cells treated with different concentrations of CAT for 48 hours.


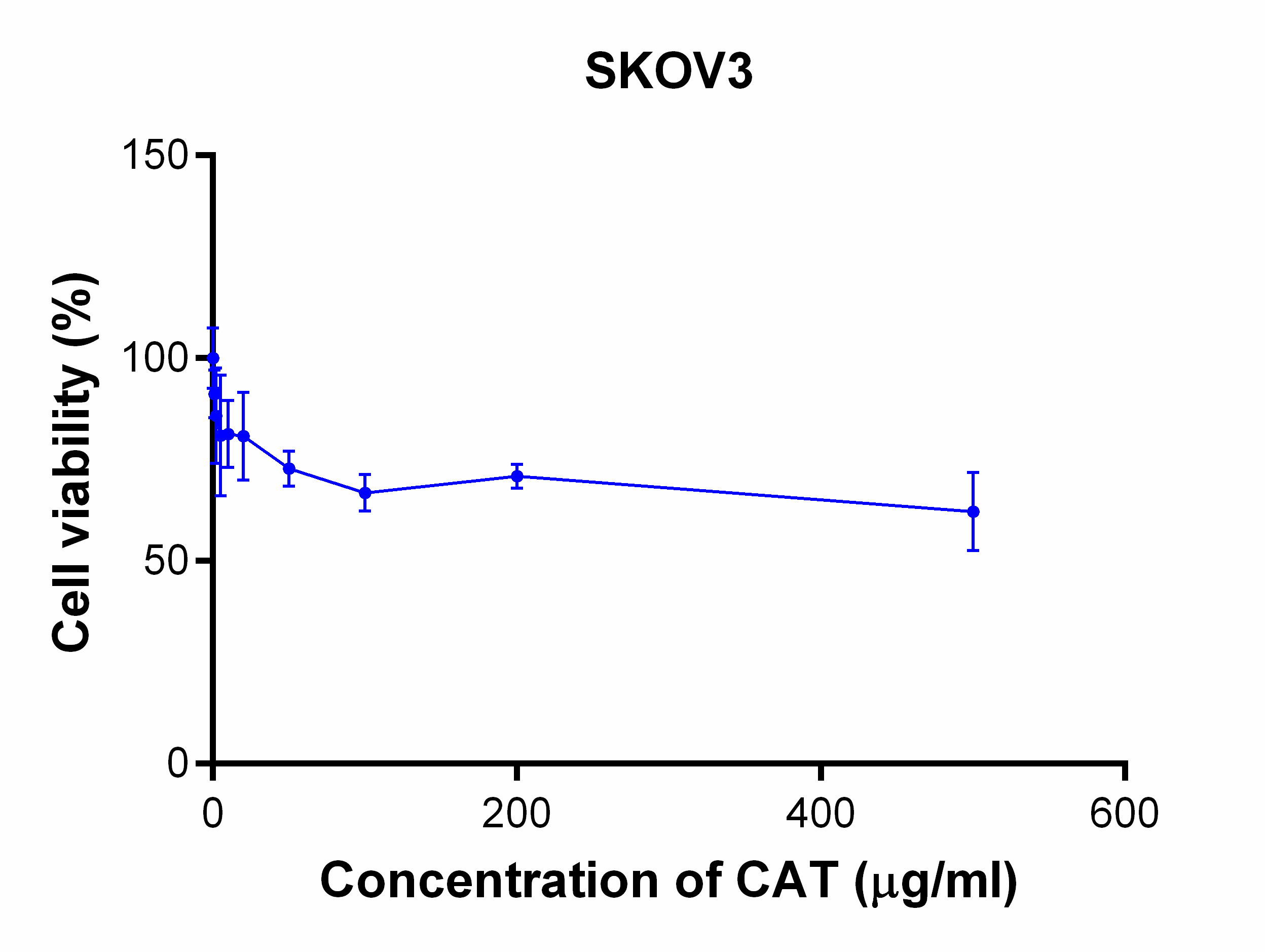


**Supplementary Figure 12.** Microfluidic devices used for Au@MSN-Ter/THPP@CM@GelMA and Au@MSN-Ter/THPP@CM@GelMA/CAT microspheres preparation.

**
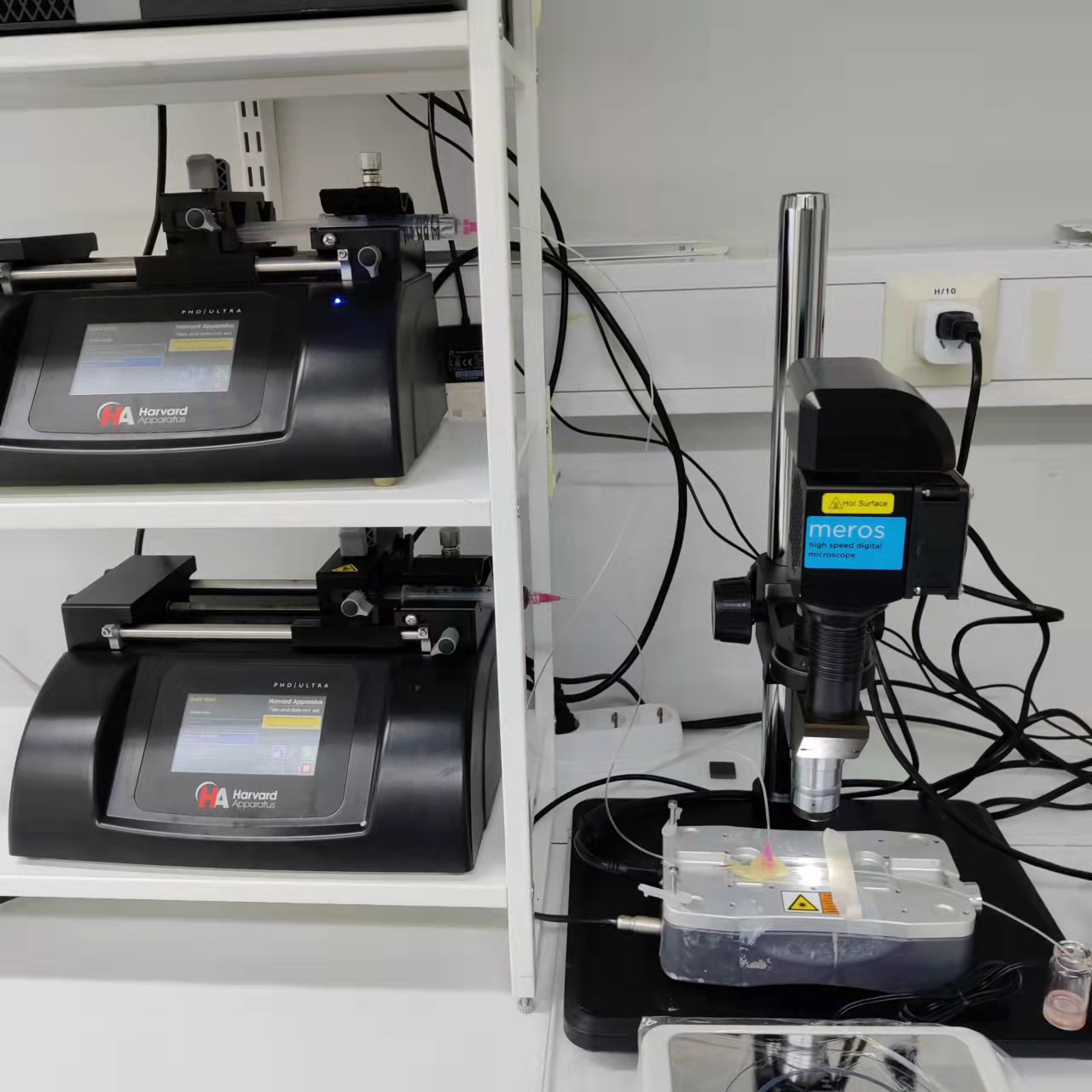
**

**Supplementary Figure 13.** Tested breaking force of different Au@MSN-Ter/THPP@CM@GelMA formulations with different concentration of Au@MSN-Ter/THPP@CM NPs.

**
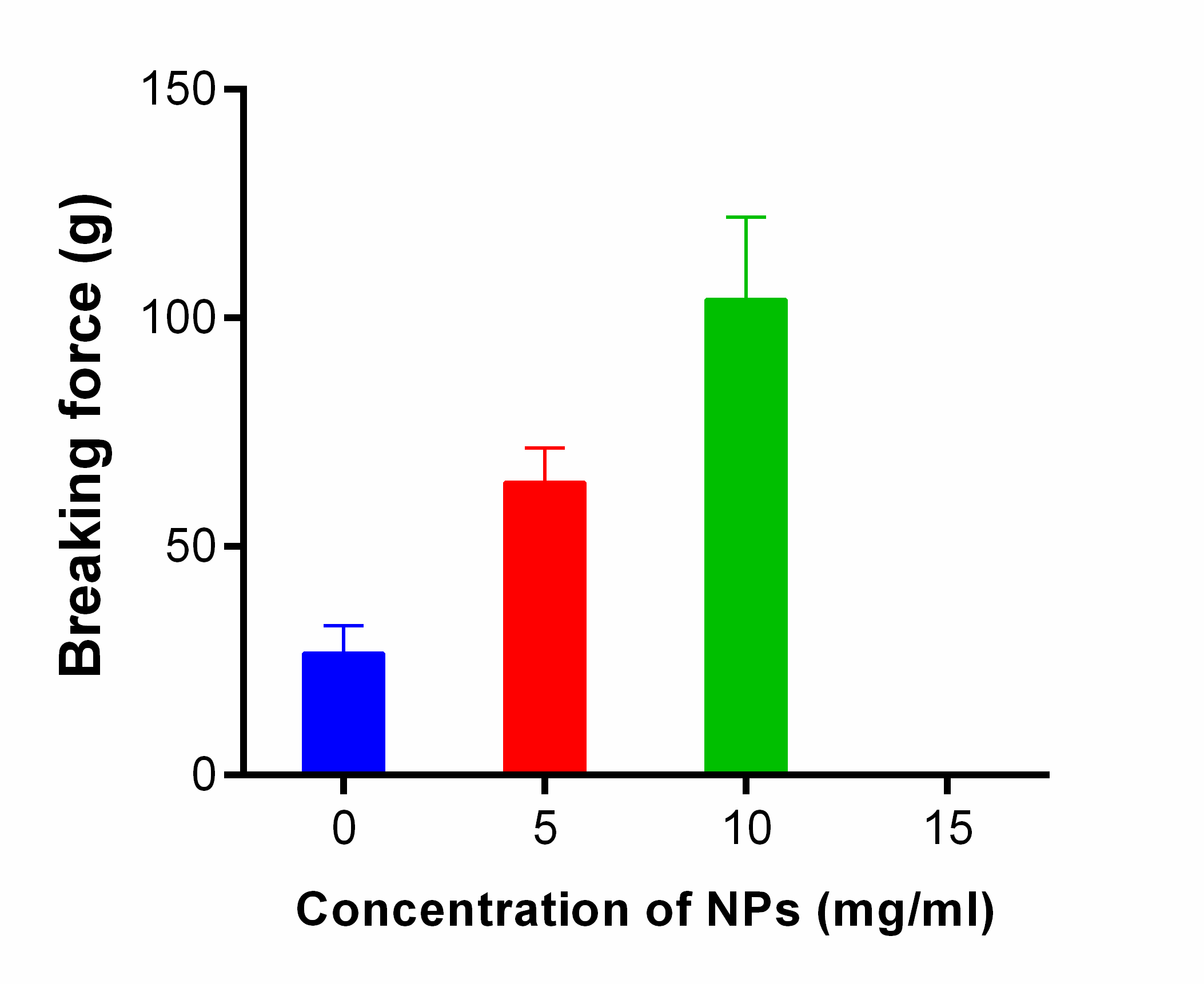
**

**Supplementary Figure 14.** Modeling of SKOV3 cell line formed orthotopic mouse ovarian cancer.

**
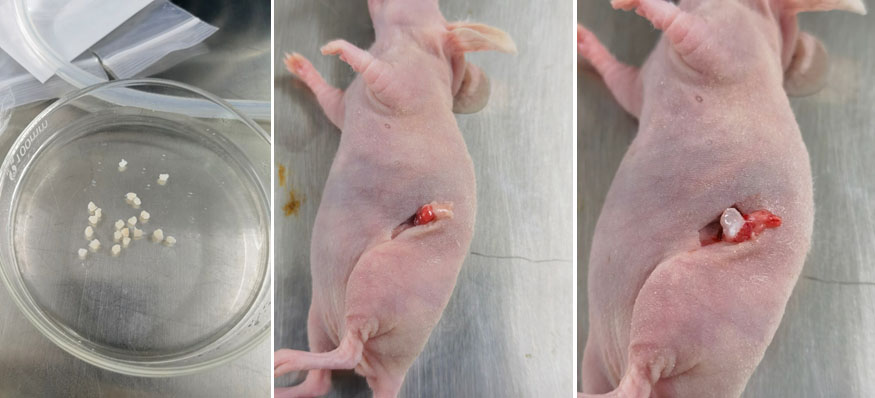
**

**Supplementary Figure15.** UV profile of Cy5.5, tested by Nanodrop 2000 (Thermo, Ma, US).


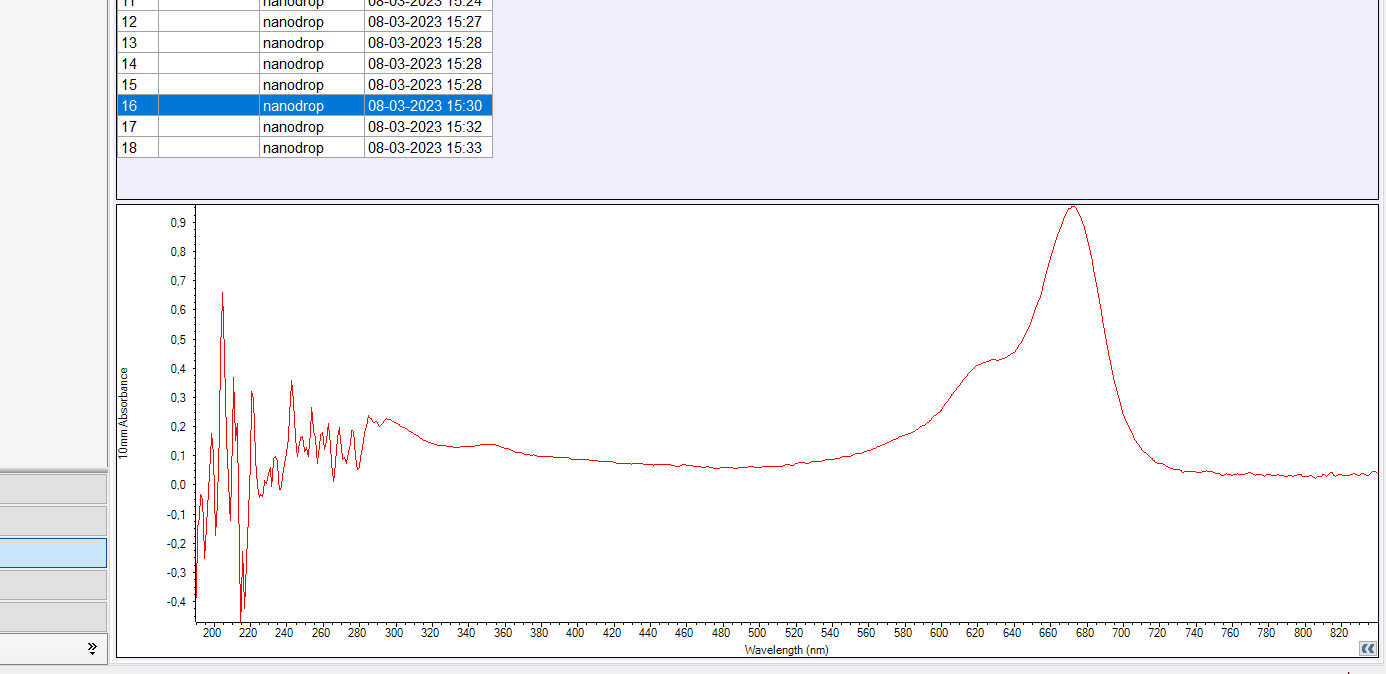


**Supplementary Figure16.** UV profile of THPP, tested by Nanodrop 2000 (Thermo, Ma, US).


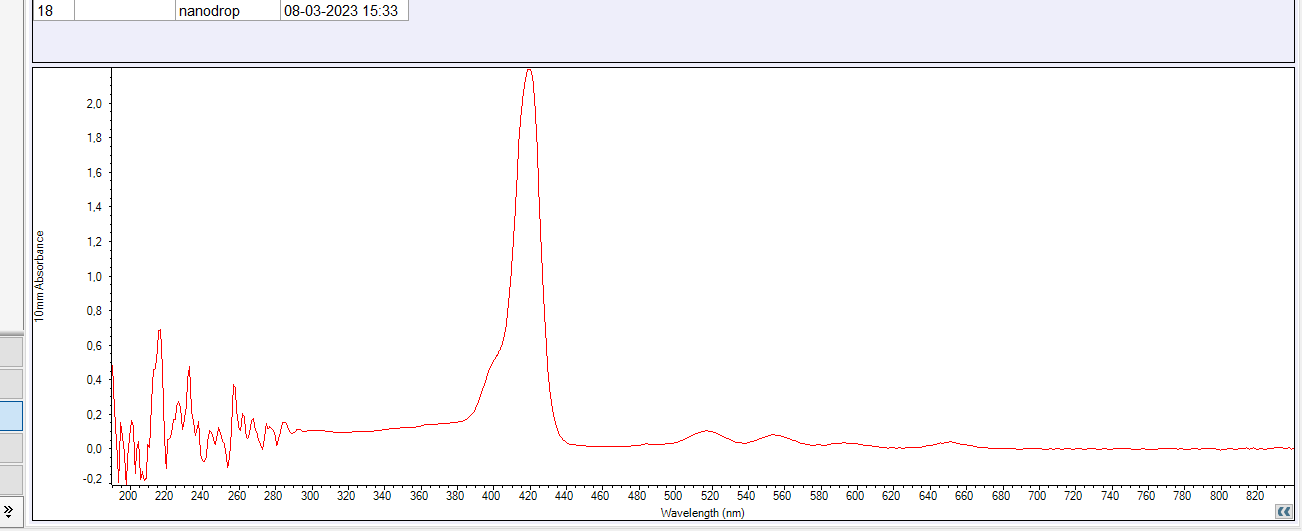


**Supplementary Figure17.** Fluorescence profile of Cy5.5, tested by Nanodrop 3000 (Thermo, Ma, US).


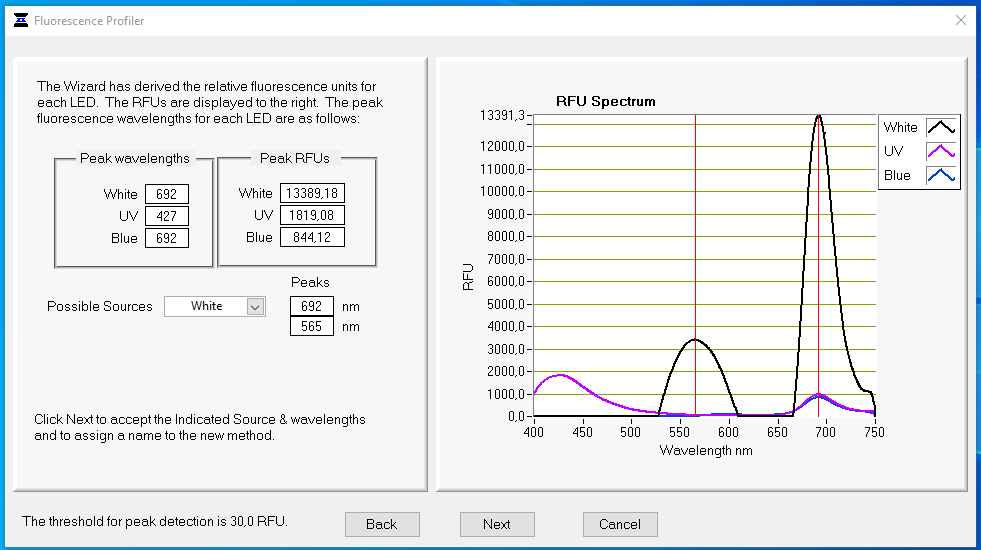


**Supplementary Figure18.** Fluorescence profile of THPP, tested by Nanodrop 3000 (Thermo, Ma, US).


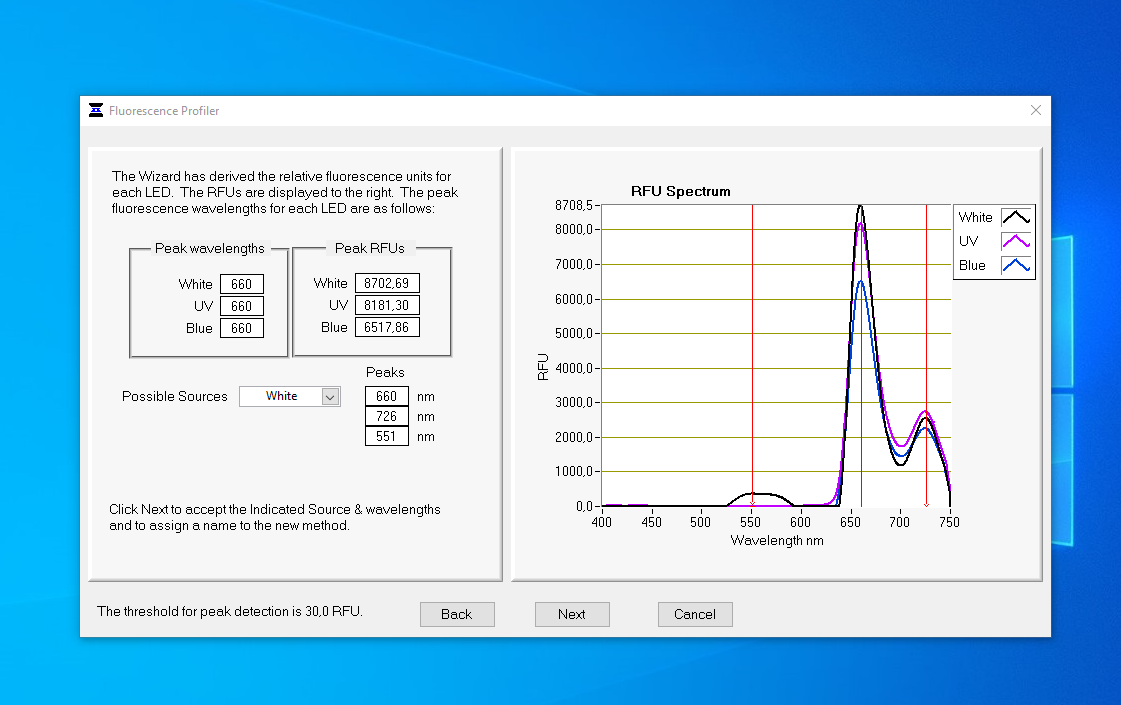


**Supplementary Figure 19.** Thermal images of SKOV3 cells transplanted orthotopic ovary cancer mice after Au@MSN-Ter/THPP@CM NPs (tail vein injection) and Au@MSN-Ter/THPP@CM@GelMA/CAT microspheres (intratumoral injection) adminstration under laser 980 irradiation (1 W/cm^2^).


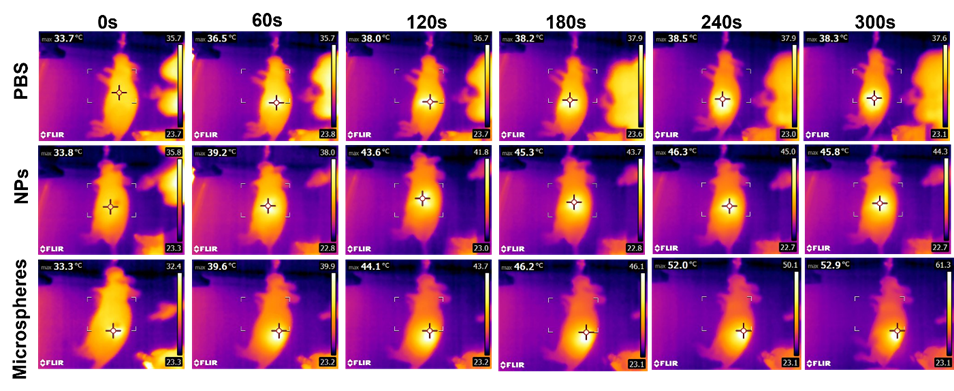


**Supplementary Figure 20.** Organ tissues THPP fluorescent signal of Au@MSN-Ter/THPP@CM NPs captured by in vivo imaging system and quantifications (From left to right and up to down panels: Heart, liver, spleen, lung, kidney, and tumor).


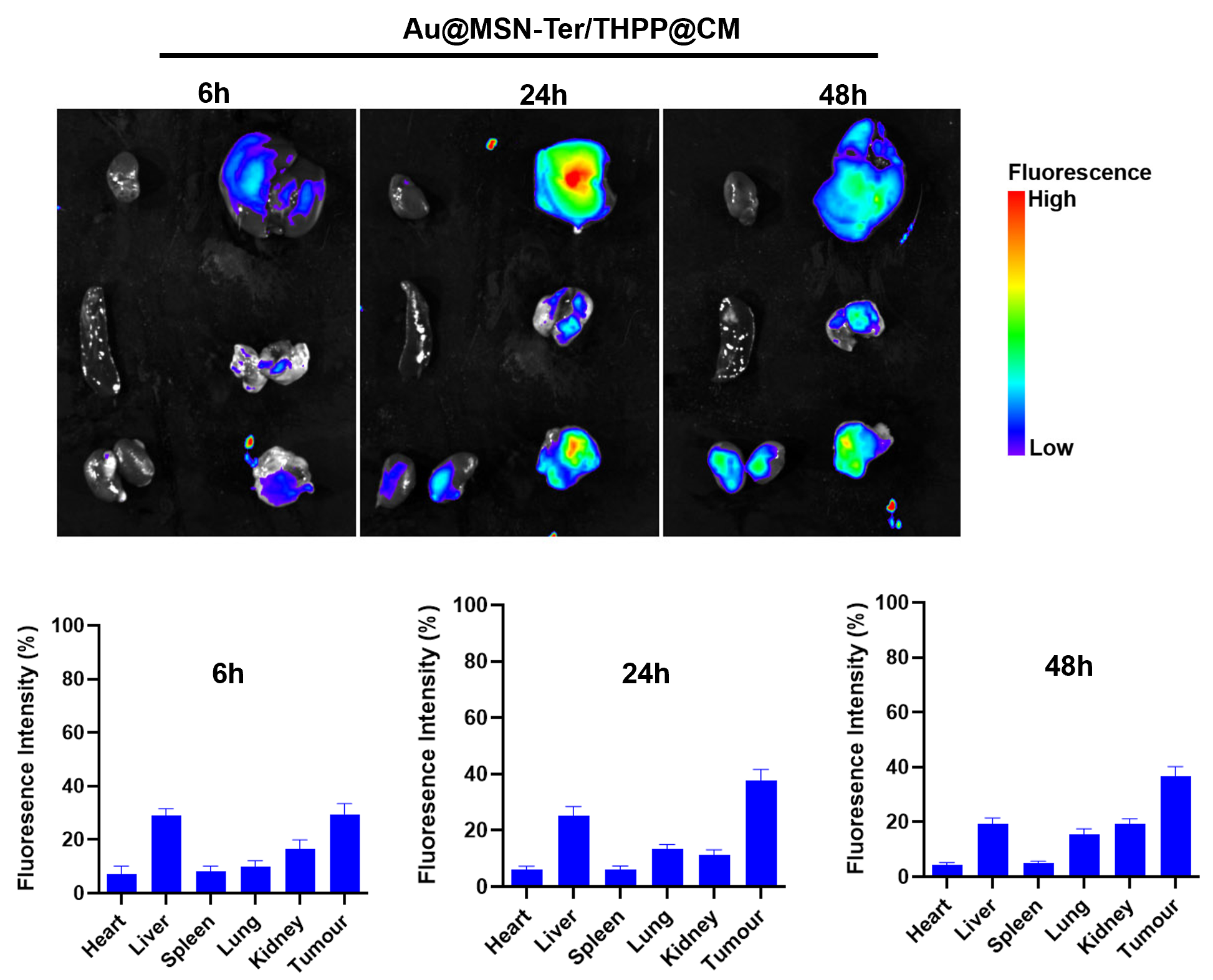

Supplement: Multimedia component 1 [file mmc1.doc]
